# Plant species richness promotes the decoupling of leaf and root defence traits while species‐specific responses in physical and chemical defences are rare

**DOI:** 10.1111/nph.20434

**Published:** 2025-02-27

**Authors:** Leonardo Bassi, Justus Hennecke, Cynthia Albracht, Marcel Dominik Solbach, Akanksha Rai, Yuri Pinheiro Alves de Souza, Aaron Fox, Ming Zeng, Stefanie Döll, Van Cong Doan, Ronny Richter, Anja Kahl, Lea Von Sivers, Luise Winkler, Nico Eisenhauer, Sebastian T. Meyer, Nicole M. van Dam, Alexandra Weigelt

**Affiliations:** ^1^ Systematic Botany and Functional Biodiversity, Institute of Biology Leipzig University Leipzig 04103 Germany; ^2^ German Centre for Integrative Biodiversity Research (iDiv) Halle‐Jena‐Leipzig Leipzig 04103 Germany; ^3^ Department of Soil Ecology Helmholtz Centre for Environmental Research – UFZ Halle 06120 Germany; ^4^ Swammerdam Institute for Life Sciences University of Amsterdam Amsterdam 1098XH The Netherlands; ^5^ Institute for Biosafety in Plant Biotechnology Julius Kühn‐Institute Quedlinburg 06484 Germany; ^6^ Terrestrial Ecology Group, Institute of Zoology University of Cologne Cologne 50674 Germany; ^7^ Department of Biogeochemical Processes Max Planck Institute for Biogeochemistry Jena 0774526 Germany; ^8^ Research Unit Comparative Microbiome Analysis Helmholtz Zentrum München Neuherberg 85764 Germany; ^9^ TUM School of Life Science, Chair of Environmental Microbiology Technische Universität München Freising 85354 Germany; ^10^ Environment, Soils and Land Use Teagasc, Johnstown Castle, Co Wexford Y35HK54 Ireland; ^11^ Institute of Biodiversity University Jena Jena 07743 Germany; ^12^ Université de Bordeaux INRAE, BFP, UMR 1332 Villenave d'Ornon 33140 France; ^13^ Plant Physiology Unit, Life Sciences and Systems Biology Department University of Turin Torino 10123 Italy; ^14^ Experimental Interaction Ecology, Institute of Biology Leipzig University Leipzig 04103 Germany; ^15^ Terrestrial Ecology Research Group, School of Life Sciences Technical University Munich Freising D‐85354 Germany; ^16^ Leibniz Institute of Vegetable and Ornamental Crops (IGZ) Großbeeren 14979 Germany

**Keywords:** biodiversity, chemical defences, fine root, functional traits, leaf, physical defences, untargeted metabolome

## Abstract

- The increased positive impact of plant diversity on ecosystem functioning is often attributed to the accumulation of mutualists and dilution of antagonists in diverse plant communities. While increased plant diversity alters traits related to resource acquisition, it remains unclear whether it reduces defence allocation, whether this reduction differs between roots and leaves, or varies among species.
- To answer these questions, we assessed the effect of plant species richness, plant species identity and their interaction on the expression of 23 physical and chemical leaf and fine root defence traits of 16 plant species in a 19‐yr‐old biodiversity experiment.
- Only leaf mass per area, leaf and root dry matter content and root nitrogen, traits associated with both, resource acquisition and defence, responded consistently to species richness. However, species richness promoted a decoupling of these defences in leaves and fine roots, possibly in response to resource limitations in diverse communities. Species‐specific responses were rare and related to chemical defence and mutualist collaboration, likely responding to species‐specific antagonists' dilution and mutualists' accumulation.
- Overall, our study suggests that resource limitation in diverse communities might mediate the relationship between plant defence traits and antagonist dilution.

## Introduction

Biodiversity is vital for the functioning of an ecosystem and its services to humanity (Cardinale *et al*., 2012; Tilman *et al*., 2014; Isbell *et al*., 2017). Researchers have demonstrated a strong increase over time in the positive relationship between biodiversity and ecosystem functioning (BEF), both in forests (Guerrero‐Ramírez *et al*., 2017; Huang *et al*., 2018) and in grasslands (Cardinale *et al*., 2007; Reich *et al*., 2012; Ravenek *et al*., 2014; Meyer *et al*., 2016; Wagg *et al*., 2022), suggesting greater importance of biodiversity than previously presumed from shorter‐term studies (Eisenhauer *et al*., 2019). Over the past decade, we have accumulated evidence that multitrophic interactions, particularly plant–soil feedback, are important drivers of the strengthening biodiversity‐functioning relationships over time (Kulmatiski *et al*., 2012; Eisenhauer *et al*., 2012b; van der Putten *et al*., 2013; Thakur *et al*., 2021). The two most likely mechanisms through which biotic interactions influence BEF relationships are the dilution of antagonists (Maron *et al*., 2011; Schnitzer *et al*., 2011; Wang *et al*., 2023; Mahon *et al*., 2024) and the accumulation of plant mutualists (van der Heijden *et al*., 1998) with increasing diversity. However, it is still unknown whether changes in antagonists and mutualists impose differing selective pressures on plants along diversity gradients (Stamp, 2003) and allow plants to reduce resource allocation to defence to promote growth in more diverse communities. Using a trait‐based approach, we aim to test how plant defences change in response to the dilution of antagonists and the accumulation of mutualists along a long‐term plant diversity gradient in experimental grasslands.

Despite the consensus that the negative effects of below‐ and aboveground antagonists decrease, while the positive effects of mutualists increase along diversity gradients, studies reveal inconsistencies (Halliday & Rohr, 2019). For example, while the dilution of antagonists appears to affect primarily specialists (Mommer *et al*., 2018; Wang *et al*., 2023), possibly due to host dilution, (resource concentration hypothesis; Root, 1973; Civitello *et al*., 2015), generalists may benefit from increased plant diversity through expanded feeding opportunities (Keesing *et al*., 2006). Nonetheless, several studies have consistently shown that antagonist pressure decreases regardless of specialisation. This was shown for soil antagonists, such as soil‐borne fungal pathogens (Mills & Bever, 1998; Yang *et al*., 2015; Wang *et al*., 2019), root‐feeding nematodes (Cortois *et al*., 2017; Dietrich *et al*., 2021) and arthropods (Amyntas *et al*., 2025), as well as for aboveground antagonists, such as arthropods and pathogens (Mitchell *et al*., 2002; Rottstock *et al*., 2014; Muiruri *et al*., 2019; Strauss *et al*., 2024). This effect may be attributed to enhanced top‐down predator control on herbivores, which can impact both generalists and specialists (Barnes *et al*., 2020; Amyntas *et al*., 2025). Similarly, the positive effect of mutualists, such as mycorrhizal fungi, biocontrol bacteria and decomposers, seems independent of specialisation (van der Heijden *et al*., 1998; Latz *et al*., 2012; Eisenhauer *et al*., 2012a). Overall, these findings suggest that antagonistic pressure on plants decreases, while support from mutualists increases with plant diversity.

Studies further suggest that this dilution of antagonists and accumulation of mutualists along plant diversity gradients may intensify over time (Eisenhauer *et al*., 2019). However, recent research found limited support for such temporal changes for belowground antagonists (Amyntas *et al*., 2025) and mutualists (Albracht *et al*., 2024). This implies that initial shifts in soil communities in response to plant diversity may stabilise relatively quickly (Eisenhauer *et al*., 2012b), maintaining belowground antagonist pressure relatively constant over the years. On the contrary, Bröcher *et al*. (2024) found that the relationship between aboveground herbivores and plant diversity is highly variable over time, suggesting that aboveground antagonists may be more susceptible to seasonal and interannual biotic and abiotic fluctuations than belowground antagonists (De Deyn & Van der Putten, 2005). These fluctuations may lead to periodic die‐offs of aboveground antagonists, potentially hampering their dilution and thus selection pressure in diverse plant communities. Furthermore, aboveground antagonists generally exert milder pressure on plants than belowground antagonists (Johnson *et al*., 2016b). Thus, belowground antagonists may be a stronger and more constant driver of plant productivity than aboveground ones.

**Fig. 1:**
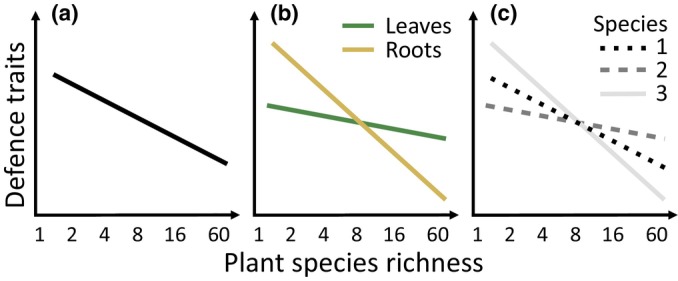
Graphical illustration of the hypothesised reduction in defence traits along the plant diversity gradient (a), a stronger response of fine root compared with leaf defence traits (b) and species‐specific responses of three hypothetical species (1, 2 and 3) (c).

Given that differing selection pressures along plant diversity gradients are known to promote changes in plant phenotypes and genotypes over time (Miehe‐Steier *et al*., 2015; van Moorsel *et al*., 2018, 2019), changes in antagonists and mutualists along plant diversity gradients likely lead to changes in plant resource allocation to defence. Based on the assumption that there may be trade‐offs between growth and defence (Stamp, 2003; Lind *et al*., 2013; Cappelli *et al*., 2020; Zaret *et al*., 2024) and that growth is closely related to plant fitness, a dilution of antagonists and an accumulation of mutualists in more diverse plant communities would reduce the need for investment into defence (Fig. 1a). Considering that Bassi *et al*. (2024) identified a stronger influence of belowground mechanisms on long‐term monoculture plant performance, we also postulate that the effect of plant diversity on root defences may be stronger than on leaves (Fig. 1b). Furthermore, while Mraja *et al*. (2011) observed a reduction in some leaf chemical defences along a plant diversity gradient for *Plantago lanceolata* L., it remains unclear whether this phenomenon can be generalised across multiple species (Fig. 1c).

Plant defence traits, that is functional traits that promote plant fitness in the presence of antagonists relative to the absence of antagonists (Didiano *et al*., 2014), are often divided into physical and chemical defences (Table 1; Moore & Johnson, 2017). Physical defences are those that deter herbivores from feeding on plant tissues through morphological or anatomical traits (Hanley *et al*., 2007), while chemical defences encompass traits related to the tissue's nutritional quality (Mattson, 1980; Poorter *et al*., 2004) and defensive phytochemicals (Raguso *et al*., 2015). Another strategy to counteract antagonists is collaboration with mutualists. Along the recently defined ‘root economics space’ (Bergmann *et al*., 2020), this is captured by the ‘collaboration gradient’, represented by a trade‐off between specific root length (SRL) and root diameter (RD), which is related to the root colonisation by mycorrhizal fungi. Despite mycorrhizal fungi enhancing plant physical defence by limiting antagonist access to roots through competition for space and resources (Rasmann *et al*., 2011), they may also promote plant chemical defences by inducing the production of defensive metabolites (Jung *et al*., 2012; Cameron *et al*., 2013; Frew *et al*., 2022).

**Table 1:** Leaf and fine root defence traits examined in this study.

Tissue	Traits	Defence correlation	Defence type	Defence mechanisms	References
Leaf	Water repellency	+	Physical defences	Surface barrier: reduced attachment and mobility of antagonists	Hanley *et al*. (2007), Gorb & Gorb (2017)
	Hair density	+			
	Toughness	+		Mechanical strength	Poorter *et al*. (2004), Hanley *et al*. (2007), Johnson *et al*. (2010), Loranger *et al*. (2012), Schuldt *et al*. (2012), Caldwell *et al*. (2016), Moore & Johnson (2017), Bröcher *et al*. (2023)
	LMA	+			
Leaf/fine root	DMC	+			
	SRL	−	
Fine root	Diameter	+		Protection through AMF^†^ (root collaboration gradient)	Rasmann *et al*. (2011), Jung *et al*. (2012), Cameron *et al*. (2013), Cortois *et al*. (2016), Johnson *et al*. (2016a), Bergmann *et al*. (2020), Frew *et al*. (2022)
	MC	+			
	
Leaf / fine root	Nitrogen	−	Chemical defences	Palatability: the nutritional quality of the tissue	Mattson (1980), Poorter *et al*. (2004)
	Alkaloids	+		Toxicity; Inducing or priming plant resistance or tolerance against plant antagonists (pathogens and insects)	Hol *et al*. (2004), Steppuhn *et al*. (2004), Alves *et al*. (2007), Nuringtyas *et al*. (2014), Dugé de Bernonville *et al*. (2017)
	Terpenoids	+			Marak *et al*. (2002), Nakashita *et al*. (2003), Zhang *et al*. (2015), Zahid *et al*. (2017), Lackus *et al*. (2018), Murata *et al*. (2019)
	Shikimates	+			Alonso *et al*. (2009), Koskimäki *et al*. (2009), Hölscher *et al*. (2014), Prasannalaxmi & Rani (2016), Olivier *et al*. (2018), Lea *et al*. (2021), Grover *et al*. (2022), Zhang *et al*. (2022), Yang *et al*. (2023)

The table reports the tissues (leaf or fine root), the traits and the defence correlation (i.e. the expected positive or negative correlation between trait and defence). As we hypothesise defence to be negatively correlated with diversity, the hypothesised trait–diversity relationships are exactly reversed. The table further reports the classification in defence type (physical or chemical), the corresponding defence mechanisms and relevant references. For ‘Alkaloids’, ‘Terpenoids’, and ‘Shikimates and phenylpropanoids’, we refer to the chemical compounds within those chemical pathways (detailed in Table S2). It is important to note that this table is based on studies using targeted metabolome quantification of one group of compounds within those pathways, while our study employs a broader untargeted metabolomic approach to assess phytochemical richness or the sum of range‐scaled feature intensity within these pathways. DMC, dry matter content; LMA, leaf mass per area; MC, mycorrhizal colonisation; Shikimates, Shikimates and phenylpropanoid; SRL, specific root length. Part of this table is derived from Bassi *et al*. (2024).

^†^ While we have classified mycorrhizal colonisation as a physical defence, we acknowledge that mycorrhizas may also promote plant chemical defences through priming.

Plant defence traits provide a framework that enables us to investigate how plant resource allocation to defence changes along plant diversity gradients. Similarly, functional traits, related to collaboration with mutualists, enable us to investigate plant investment in mutualistic interactions. For instance, some leaf and root traits related to physical and chemical defences, such as leaf mass per area (LMA), leaf toughness, nitrogen and phenolics content, and metabolome, as well as traits related to collaboration with mutualists, such as SRL, have been shown to change along plant diversity gradients (Scherling *et al*., 2010; Mraja *et al*., 2011; Ristok *et al*., 2019; Peng & Chen, 2021; Weinhold *et al*., 2022; Felix *et al*., 2023). Although several of these traits are also related to resource acquisition (Weigelt *et al*., 2021), their changes often affect plant–antagonist and plant–mutualist interactions (Muiruri *et al*., 2019; de Vries *et al*., 2021; Bröcher *et al*., 2023; Ristok *et al*., 2023b). However, plant species frequently deploy different types of defences: While grasses are often defended through physical defences, forbs are more commonly defended through chemical defences (Eichenberg *et al*., 2015; Bassi *et al*., 2024). Furthermore, plant–antagonist interactions are also affected by associational effects, where the herbivory rate experienced by a focal plant species depends on the characteristics, such as defence traits, of the surrounding plant community and herbivores' feeding preferences, leading to either associational susceptibility or resistance (Barbosa *et al*., 2009; Underwood *et al*., 2014). For example, the ‘neighbour contrast susceptibility and defence’ hypothesis (Alm Bergvall *et al*., 2006) suggests that if a focal plant species is better defended than the surrounding plant community, it will experience lower herbivory compared with when it grows among plants that are better defended than itself. Indeed, changes in herbivory rates along plant diversity gradients have been shown to differ between plant species in magnitude and direction (Bröcher *et al*., 2023). It is thus reasonable to hypothesise that changes in defences along plant diversity gradients are species‐specific (Fig. 1c). Indeed, despite consistent trait–diversity relationships among plant species (Roscher *et al*., 2018), species‐specific responses have also been observed (Gubsch *et al*., 2011; Roscher *et al*., 2011b; Lipowsky *et al*., 2015).

In this study, we measured for the first time a comprehensive set of physical and chemical defence traits in leaves and fine roots (summarised in Table 1) of 16 grassland plant species growing along a 19‐yr‐old plant species richness gradient in the Jena Experiment (Roscher *et al*., 2004). We hypothesised that the observed dilution of antagonists and accumulation of mutualists along plant species richness gradients would trigger:

1. a reduction in plant defence traits (Fig. 1a);
2. a more pronounced defence reduction in fine roots compared with leaves (Fig. 1b); and
3. species‐specific defence responses (Fig. 1c).

## Materials and Methods

### Study site and experimental design

The investigation took place within the Jena Experiment, a grassland biodiversity experiment initiated in 2002 (Roscher *et al*., 2004). The Experiment is situated in the floodplain of the Saale River near Jena, Germany (latitude: 50.95, longitude: 11.62, altitude: 130 m above sea level), a region with a mean annual air temperature of 9.9°C and annual precipitation of 660 mm (Hoffmann *et al*., 2014). The experimental site had formerly been an arable field for 40 yr. The experiment consists of 80 experimental plant communities representative of the Arrhenatherion mesophilic grassland (Ellenberg, 1988), with varying species and functional richness. The six species richness (SR) levels, 1, 2, 4, 8, 16 and 60, are nearly orthogonally crossed with the four functional richness levels consisting of grasses, legumes, small herbs and tall herbs (Roscher *et al*., 2004). Each SR level had 16 replicates (except for the monocultures with 14 and the 60‐species mixtures with 4 replicates). Species compositions were randomly determined. In addition, monocultures of all 60 species were established. Plots are arranged in four blocks to account for variations in soil texture caused by distances to the river. Plots were mown twice a year in June and September, following the typical extensive meadow management of the region, and were not fertilised during the experimental period. Weeding occurred at least twice per growing season. Additional details on the experiment are reported in Roscher *et al*. (2004).

### Field sampling of leaf and fine root samples

Sixteen species (Table S1), four of each functional group, were selected and harvested at peak biomass from 17 to 31 May 2021 in all plots where they were present (totalling 128 species per plot combinations). Due to the randomness of species selection in the experimental design and the local extinction of certain species in specific plots, not all species were uniformly represented across the diversity gradient. In addition, the number of sampled species per plot combination varied depending on the species (refer to Table S1 for detailed species occurrences).

In each plot, we sampled above‐ and belowground parts of three individuals per species. An additional one or two individuals were harvested for small species to collect enough leaf and fine root material for chemical analyses. The 428 sampled individuals were randomly selected and marked in early spring, avoiding plot edges. The aboveground plant part was harvested by cutting the stem 1–2 cm above the ground and placed in a sealed plastic bag along with a wet paper towel to rehydrate the leaves to their full potential (Pérez‐Harguindeguy *et al*., 2013). We sampled the roots of the same individuals by extracting a soil core with a depth of 10 cm and a diameter of 6.9 cm. All collected samples were quickly put into a cooling box and transferred to a 4°C refrigerator within 6 h. Samples were processed from 6 to 26 h after sample collection. Subsamples for the metabolome analysis were always collected within 14 h after field collection.

### Leaf sample preparation and morphological trait measurements

All leaf morphological and chemical trait measurements were performed on fully expanded, undamaged and nonsenescent leaves excluding petioles and rachis. The leaves of each individual were divided into three subsamples according to their position (internode). For grasses without flowering stems, this was not possible, and random leaves were taken instead. One subsample comprising one or three leaves or leaflets (depending on leaf size) collected from the second or third internode from the top was frozen in liquid nitrogen and stored at −80°C to be used for the untargeted metabolome analysis. A second subsample consisting of one to five leaves (depending on leaf size) from the third to the fifth internode from the top was used for morphological trait measurements. The third subsample consisted of all the remaining leaves, pooled at the species per plot level, was frozen in liquid nitrogen and stored at −80°C for nitrogen quantification.

Leaf morphological traits were measured at the individual level and then averaged at the species per plot level. Leaves were first weighed and scanned with a flatbed Epson Expression 11000XL scanner at 600 DPI (Epson, Tokyo, Japan). After the scan, one leaf per individual was used for water repellency (WR; deg.), hair density (no. of hairs mm^−2^) and toughness (N mm^−1^; the ratio of maximum shearing force to thickness) measurements (Pérez‐Harguindeguy *et al*., 2013). Leaf images for determining WR and hair density were analysed in imagej (v.1.53a; Schneider *et al*., 2012). During the storing or handling process, five samples were damaged, resulting in missing WR measurements for those samples. Leaves were weighed after being oven‐dried at 70°C for 72 h. We determined LMA (g m^−2^) as the ratio of dry weight to leaf area and leaf dry matter content (LDMC; g g^−1^) as the ratio of dry to fresh weight (Pérez‐Harguindeguy *et al*., 2013). Further details on the leaf morphological trait measurements are reported in Methods S1.

### Fine root sample preparation, morphological trait and mycorrhizal colonisation measurements

Before proceeding with the root washing, soil cores were soaked in cold water for 15 min. Roots were washed carefully inside a water‐filled bucket to prevent root damage. We repeatedly refreshed the water by filtering out soil debris until all roots were free from soil. We collected only roots attached to the stem of the target individual and discarded all the rest. For further analyses, we retained all fine roots with a diameter lower than 2 mm. A random subsample of the fine roots of each individual was collected, frozen in liquid nitrogen and stored at −80°C to be used for the untargeted metabolome analysis. The remaining fine roots of each individual were pooled at the species per plot level. From this bulked sample, we extracted a random subsample for morphological trait measurements and a random subsample for the estimation of mycorrhizal colonisation (MC). All remaining fine roots were frozen in liquid nitrogen and stored at −80°C to be used for nitrogen quantification.

To assess fine root morphological traits, the bulked subsample was scanned with a flatbed scanner at 600 DPI (Expression 11000XL; Epson, Düsseldorf, Germany) and weighed after being carefully dried with a paper towel. Fine roots were then freeze‐dried to estimate the dry weight. We retrieved mean RD (mm) and root length from the scans using rhizovision (v.20.0.3; Seethepalli *et al*., 2021). We estimated SRL (m g^−1^) as the ratio of root length to root dry weight and root dry matter content (RDMC; g g^−1^) as the ratio of dry to fresh weight (Freschet *et al*., 2021). Four SRL, three RDMC and three RD observations were excluded due to contamination of roots from other species, the inclusion of roots with a diameter larger than 2 mm, loss of sample material before the dry weight measures, or missing scan.

We measured the MC rate (%) using the method developed by Trouvelot *et al*. (1986). Fine roots were first rinsed in water and destained by incubation in a 10% KOH solution at 95°C for 10 min. We stained the roots with an acidic ink solution (5% acetic acid and 5% Pelican blue ink). We removed the excess by rinsing them in water and incubating them twice at 75°C in a 20% acetic acid solution. Roots were mounted on microscope slides for analysis with a digital microscope (Keyence VHX, Osaka, Japan) to collect at least nine images per sample at 200× magnification. The images were used to estimate the MC rate in six cover classes as described in Trouvelot *et al*. (1986). Cover classes were converted to percentages and all replicates averaged to the plot level. For some samples, the destaining procedure did not work properly. This resulted in 21 missing data points: four for *Arrhenaterum elatius* L., *Geranium pratense* L. and *Vicia cracca* L.; three for *Lotus corniculatus* L.; two for *Galium mollugo* L. and *Taraxacum officinale* L.; and one for *Medicago × varia* Martyn and *Plantago media* L.

### Nitrogen measurements

We estimated leaf and root relative nitrogen content (N; % of dry weight) by combining two methods to reduce laboratory workload and costs. For 59% of the samples, we used 10 mg of milled material and an elemental analyser (VarioEL II, Elementar, Hanau, Germany). For the remaining 41% of the samples, we predicted nitrogen content with a bootstrapped CARS‐PLSR model (Richter & Bassi, 2023) using near‐infrared spectral data collected with a Multi‐Purpose FT‐NIR‐Analyzer (MPA, Bruker Corp., Billerica, MA, USA). The model was calibrated with spectral data from this experiment and published data (Bassi *et al*., 2023) and achieved excellent prediction accuracy in internal model validation (*R* ^2^ = 98%, RMSE = 0.16%, RPD = 7.44). Details of the measurements are given in the Methods S2.

### Untargeted metabolomics analyses

The untargeted metabolome analysis was performed on nonvolatile, polar, semipolar and apolar metabolites between 100 and 1600 Dalton, extracted from leaf and fine root with a solution comprising 75% methanol and 25% water acetate buffer using an ESI‐UHR‐Q‐ToF‐MS (maXis impact, Bruker Daltonics, Hamburg, Germany) in positive mode, following the procedure described in Weinhold *et al*. (2022) with minor modifications (Bassi *et al*., 2024). Sample preparation included freeze‐drying, manual homogenisation with scissors, and milling in 2‐ml tubes with ceramic beads. The obtained raw data were processed in Bruker Compass MetaboScape Mass (2022b v.9.0.1; Build 11 878; Bruker Daltonics, Hamburg, Germany) using MetaboScape's T‐ReX algorithm.

Raw data processing was performed on leaf and root together. This caused features with high‐intensity values in one tissue type to appear at low‐intensity in the other. To minimise the influence of low‐intensity features, we applied the same MetaboScape feature filtering criteria to the leaf and root datasets separately in R. The entire process, including sample extraction, LC‐MS analysis, MetaboScape settings and feature filtering, is reported in the Method S3.

After removing features derived from blanks and internal standards (1177 features) and applying the feature filtering at the tissue level, the dataset included 10 667 features of which 9551 with an MS/MS fragmentation pattern. For these features, we performed a de‐novo feature annotation and classification based on the MS/MS fragmentation pattern with sirius v.5.7.2 (Dührkop *et al*., 2019). Annotations were re‐ranked with ZODIAC (Ludwig *et al*., 2020), and molecular structures were assigned with CSI:FingerID (Dührkop *et al*., 2015; Hoffmann *et al*., 2021) and classified using the deep neural network‐based natural product classifier (NPClassifier; Kim *et al*., 2021). We used 5 ppm accuracy and limited the element to CHNPOS and left the other settings to default for SIRIUS and ZODIAC. For CSI:FingerID, formulas from all databases were selected. We discharged features with a ZODIAC score lower than 0.5 and NPC pathway probability lower than 0.5.

### Estimation of chemical defence traits from untargeted metabolomic analysis

We used an untargeted metabolome analysis focusing on secondary metabolites to quantify chemical richness, calculated as the number of mass signals of putative metabolites, or features (hereafter feature richness), as well as the sum of the range‐scaled feature intensity (hereafter feature sum) within three major chemical defence pathways: alkaloids, terpenoids, and shikimates and phenylpropanoids (NPClassifier; Kim *et al*., 2021). The selection of these pathways was informed by an extensive literature review (reported in Table S2) and chosen due to their pivotal roles in plant defences. However, we acknowledge that compounds within those pathways are involved also in other biological processes and that our approach represents a very global estimate of the allocation to defence.

While feature richness does not provide a direct quantitative measure of defence phytochemicals, it serves as a proxy of the overall diversity and number of final products within that pathway (Müller & Junker, 2022). Plant individuals showing higher feature richness within a specific defence pathway are expected to possess enhanced defence against a broader array of antagonists compared with those with lower feature richness (Whitehead *et al*., 2021; Fernandez‐Conradi *et al*., 2022). However, the concentration of highly toxic compounds may act as a stronger deterrent than the richness of potentially less toxic compounds. To extract a more robust proxy of total allocation to defence, we summed the range‐scaled feature intensity within each selected pathway. Range scaling was calculated separately for roots and leaves. For each feature, scaling was performed by subtracting the minimum intensity value across all samples from the intensity of each sample and then dividing by the intensity range across all samples ($y_{i}=\frac{x_{i}-\min\left(x\right)}{\max\left(x\right)-\min\left(x\right)}$), and was chosen to account for discrepancies in feature intensity detection caused by differences in ionisation (Smilde *et al*., 2005). While this scaling results in the loss of absolute feature intensity information, it gives equal weight to each feature (Sun & Xia, 2024). Thus, the sum of range‐scaled intensities within a pathway does not provide an absolute measure of the total concentration of phytochemicals within a pathway (i.e. alkaloids), but it rather indicates whether the concentration of features within a pathway increases or decreases.

We derived feature richness and the sum within the three selected pathways from 428 individual leaf and 391 individual root samples, averaging the data per plot at the species level. Due to limited root material, 22 individuals could not be analysed, and 15 individuals were excluded from the analysis due to contamination from roots of other species or moss rhizoids. This resulted in a missing value in our final data for *Vicia cracca* in a 60‐species mixture.

### Statistical analysis

We performed all statistical analyses in R (v.4.3.2; R Core Team, 2023). We used principal component analysis (PCA) to evaluate the coordination of leaf and root defence traits across species, with missing values imputed by the median. We used linear mixed models (lme4 R package v.1.1–35.1; Bates *et al*., 2015) to examine the effect of plant community sown SR (log scale), plant species identity (ID) and their interaction (SR:ID) on each plant defence traits separately. The random effect consisted of the experiment plot nested within block to account for repeated measurements within each plot (multiple species per plot) and the distance of the plot to the river (block). To assess term significance, we used ANOVA type III sum of squares (lmertest R package v.3.1‐3; Kuznetsova *et al*., 2017) and adjusted *P*‐values for false discovery rate (FDR) due to multiple testing (Benjamini & Hochberg, 1995). The FDR correction was done separately for the *P*‐values relative to the three terms in the model (SR, ID and SR:ID). The beta coefficients (slopes), their confidence interval and *P*‐values of each defence trait changes along the plan SR gradient for each species were extracted from the models with the emtrends function from the emmeans R package (v.1.10.0; Lenth, 2024). To obtain standardised regression beta coefficients of each defence trait along the plant SR gradient among all species, we ran additional linear mixed models using Z‐transformed (mean‐centred and scaled) plant defence traits and only plant community sown SR (log scale) as the explanatory variable. To consider variations in species defence trait means and response to the SR gradient, ID was added as a random intercept only or as a random intercept and slope. Specifically, the random slope for ID was applied only to traits for which the SR:ID term was significant (FDR *P*‐values < 0.05) in the first set of models. The proportion of variance explained, marginal and conditional R^2^, was extracted from the models using the mumin R package (v.1.47.5; Bartón, 2023).

## Results

### Features classification

**Fig. 2:**
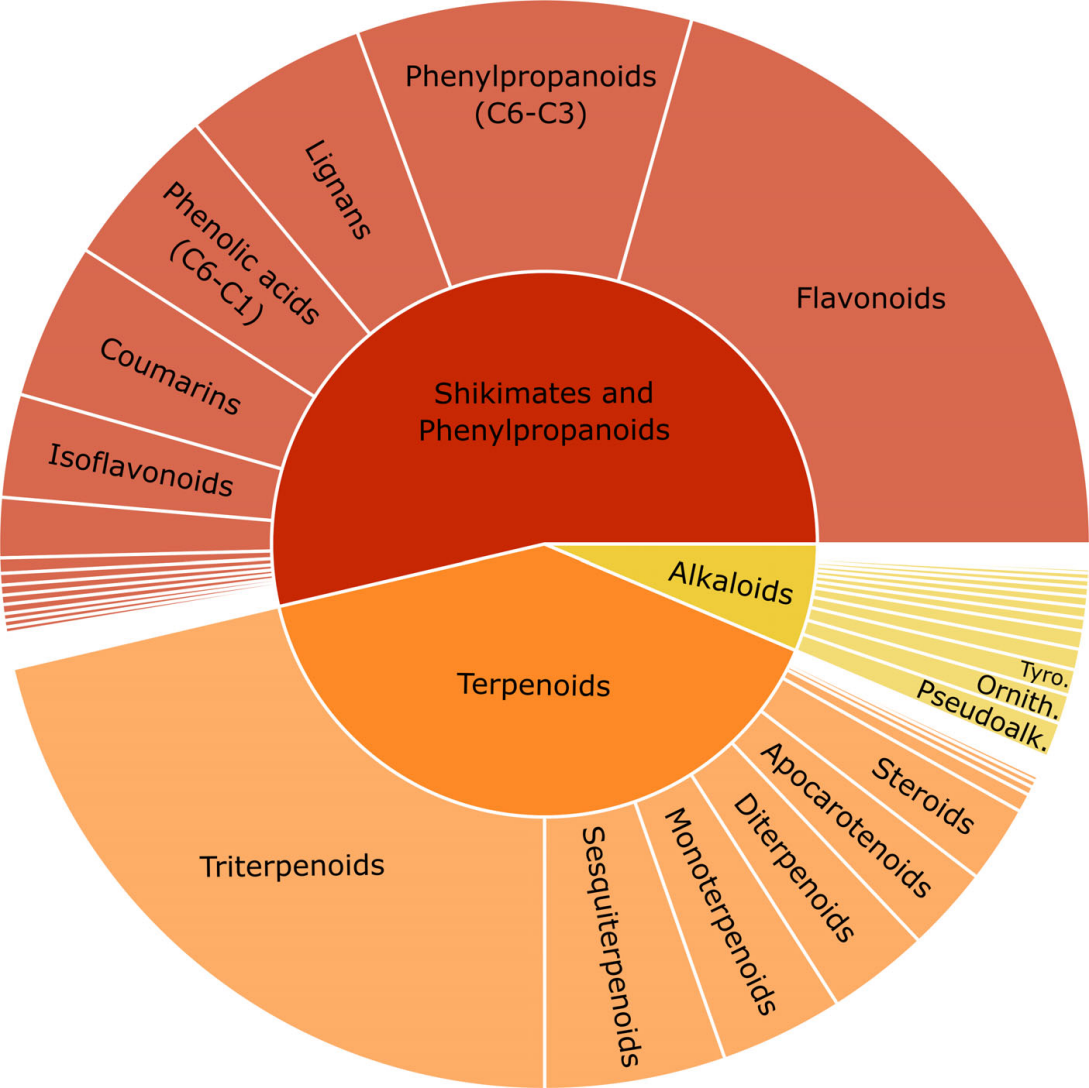
Sunburst plot showing the proportion of features for the chemical superclasses (outer circle) that could be classified with the NPClassifier within three selected pathways (inner circle; alkaloids, terpenoids and shikimates and phenylpropanoids) across all samples. Colours depict the three pathways. Only superclasses with the highest number of features within each pathway are reported. Interactive sunburst plots, including chemical classes of leaves and fine roots samples are available in the Figure S1. Ornith., Ornithine alkaloids; Pseudoalk., Pseudoalkaloids; Tyro., Tyrosine alkaloids.

Of the 9551 features with an MS/MS fragmentation pattern, 5187 (54%) were classified according to our accuracy threshold (ZODIAC score and NPC pathway probability above 0.5). Shikimates and phenylpropanoids (2173 features), terpenoids (1618 features) and alkaloids (258 features) accounted for 20, 15 and 2% of the total features, respectively (Table S3). The other pathways, amino acids and peptides, carbohydrates, fatty acids and polyketides, accounted for 2 to 3% of the total features. The superclasses with the highest number of features within shikimates and phenylpropanoids were flavonoids, phenylpropanoids, lignans, phenolic acids and coumarins. The superclasses with the highest number of features for terpenoids were tri‐, sesqui‐, mono‐ and di‐terpenoids. Within alkaloids, the superclasses with the highest number of features were pseudoalkaloids, and ornithine, tyrosine and anthranilic acid alkaloids. The detailed number of features classified within each pathway is shown in Figs 2, S1; Tables S4.

### Defence traits coordination

**Fig. 3:**
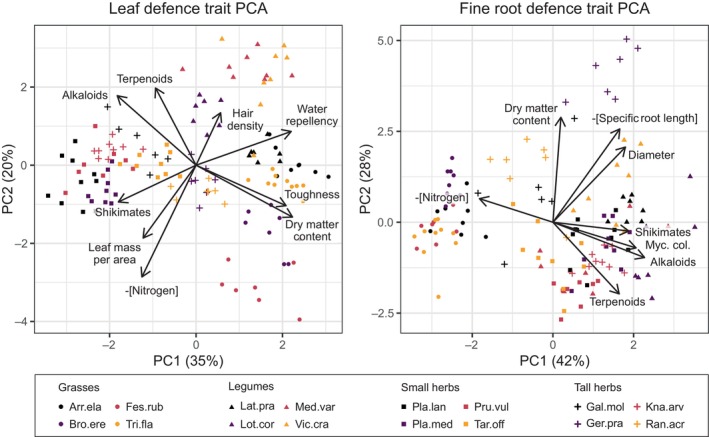
Principal component analysis (PCA) of leaf (left) and root (right) defence traits. Variance explained by each component is reported in the axis titles. Different shapes indicate plant functional groups and species within each functional group are depicted in different colours. Traits negatively correlated with defence (nitrogen and specific root length; Table 1) were multiplied by −1 to ensure that loading vectors indicate increased defence. For chemical traits related to the metabolome, alkaloids, terpenoids and shikimates, only the sum of range‐scaled feature intensity (not feature richness) was used to avoid over‐representation of chemical defences. Several traits are log‐transformed to enhance linearity among traits (Supporting Information Table S5). Shikimates, Shikimates and phenylpropanoids. Full species names: Arr.ela, *Arrhenatherum elatius* L.; Bro.ere, *Bromus erectus* Huds.; Fes.rub, *Festuca rubra* L.; Gal.mol, *Galium mollugo* L.; Ger.pra, *Geranium pratense* L.; Kna.arv, *Knautia arvensis* (L.) Coult.; Lat.pra, *Lathyrus pratensis* L.; Lot.cor, *Lotus corniculatus* L.; Med.var, *Medicago × varia* Martyn; Pla.lan, *Plantago lanceolata* L.; Pla.med, *Plantago media* L.; Pru.vul, *Prunella vulgaris* L.; Ran.acr, *Ranunculus acris* L.; Tar.off, *Taraxacum officinale* L.; Tri.fla, *Trisetum flavescens* (L.) P.Beauv.; Vic.cra, *Vicia cracca* L.

The leaf trait PCA's first two principal components (PCs) explained 35 and 20% of the variance, respectively, while those of the root PCA explained 42 and 28%, respectively (Fig. 3). The first leaf PC revealed a trade‐off between physical (toughness, dry matter content and WR) and chemical defences (shikimates and alkaloids; Table S5). The second leaf PC showed a similar trade‐off between physical (LMA) and chemical defences (alkaloids and terpenoids), although not all chemical defences (nitrogen and shikimates) followed this trade‐off.

Several root physical and chemical defences (shikimates, alkaloids, MC and RD) were positively associated along the first root PC, except for root nitrogen. The second root PC highlighted a trade‐off between physical (RDMC, RD and SRL) and chemical defences (terpenoids). Overall, the PCA analysis revealed multiple trade‐offs between physical and chemical defences.

### Species richness effects on defence traits

**Fig. 4:**
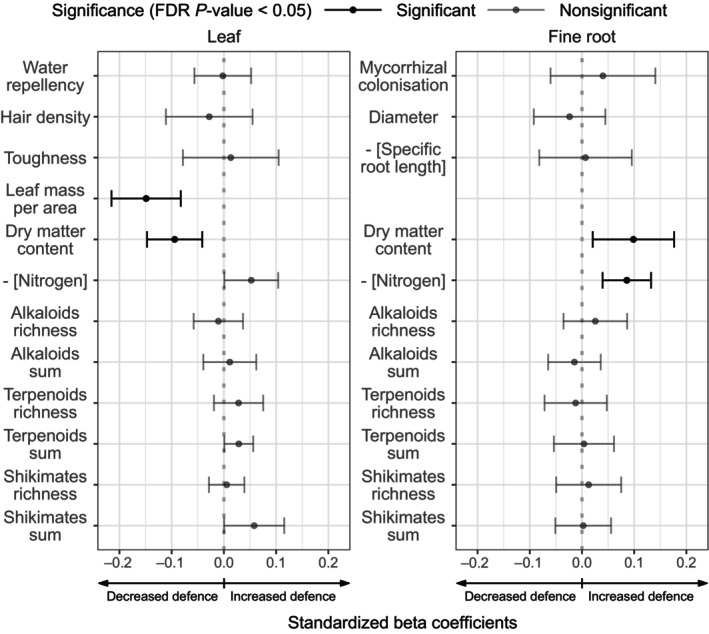
Standardised beta coefficients of plant defence traits change along the plant species richness gradient (log scale) based on linear mixed models with species as random intercept or random intercept and slope (Supporting Information Table S6; random effect structure was based on species richness (SR): species identity (ID) significance in Table 2). Standardisation was performed by running models with *Z*‐transformed defence traits (centred and scaled). Traits negatively correlated with defence (nitrogen and specific root length; Table 1) were multiplied by −1 to ensure that positive beta coefficients consistently indicate increased defence, as indicated by the arrows below each panel. Error bars show the 95% confidence interval. Significant coefficients (*P* < 0.05), based on false discovery rate (FDR)‐adjusted *P*‐values (Table 2) are reported in black, while nonsignificant ones are in grey. Leaf defence traits are shown on the left and roots on the right. Model coefficients are reported in Table S6. Shikimates, Shikimates and phenylpropanoid.

Among the 23 defence traits measured in this study, only four exhibited a significant change along the SR gradient (Table 2, column SR). With increasing plant SR, plants showed lower LMA (FDR *P*‐value = 0.0004), LDMC (FDR *P*‐value = 0.0043) and root N (FDR *P*‐value = 0.0001) compared with plants growing in low‐diversity communities (Table 2; Fig. 4; note that in Fig. 4, the coefficients for nitrogen are multiplied by −1). In contrast to LDMC, RDMC increased along the SR gradient (FDR *P*‐value = 0.0229). Similar to root N, leaf N tended to decrease along the SR gradient (Fig. 4), albeit not significantly (FDR *P*‐value = 0.2248). None of the proxies for chemical defence allocation within the three selected pathways changed significantly along the SR gradient after FDR correction (Table 2; Fig. 4). However, without FDR correction, the production of defensive phytochemicals within the terpenoids (*P*‐value = 0.0490) and shikimates and phenylpropanoids (*P*‐value = 0.0745) pathways in leaves showed a significant or marginally significant increase along the SR gradient, for the feature sum parameter of the two pathways. In summary, while two out of five leaf physical defences showed a clear decrease along the SR gradient, two out of seven leaf chemical defences tended to increase, albeit not significantly when correcting for FDR. On the contrary, one out of four root physical defences and one out of seven root chemical defences increased along the SR gradient.

**Table 2:** ANOVA table based on type III sum of squares for the linear mixed models with plant defence traits as response variable and plant species richness (log scale; SR), plant species identity (ID) and their interaction (SR:ID) as explanatory variables.

		SR		ID		SR:ID		*R* ^2^ (%)
		df	*F*‐value	df	*F*‐value	df	*F*‐value	mar/con
Leaf	Water repellency	88.195	0.073	88.884	8.107***	88.702	0.707	84/86
	Hair density	32.245	0.454	94.500	6.104***	90.723	0.502	73/77
	Toughness	94.466	0.180	94.343	1.898.	94.115	1.064	58/61
	Leaf mass per area	24.841	20.049***	92.725	12.244***	85.659	1.137	83/87
	Dry matter content	20.334	13.213**	92.431	18.241***	84.911	1.779	90/92
	Nitrogen	24.996	2.542	93.347	15.863***	88.198	0.980	87/90
	Alkaloids richness	95.435	0.028	95.128	19.035***	94.907	0.888	90/90
	Alkaloids sum	94.743	0.308	94.576	14.399***	94.327	0.341	85/86
	Terpenoids richness	27.371	1.923	91.746	45.569***	86.813	2.150*	95/97
	Terpenoids sum	95.218	3.975	94.925	48.528***	94.657	1.151	96/96
	Shikimates richness	34.334	0.538	91.888	48.456***	88.817	1.649	94/96
	Shikimates sum	28.679	3.425	85.065	17.281***	79.57	0.977	85/93
Fine root	Specific root length	24.988	0.054	90.434	4.869***	86.791	0.883	70/74
	Diameter	9.208	0.341	91.010	6.582***	84.210	0.617	76/77
	Mycorrhizal colonisation	73.274	1.243	72.906	10.184***	72.808	2.356*	81/83
	Dry matter content	92.154	7.644*	92.040	4.313***	92.051	1.837.	74/74
	Nitrogen	94.973	18.317***	94.649	18.185***	94.323	1.718	90/90
	Alkaloids richness	22.387	0.902	92.160	10.257***	86.687	1.590	82/86
	Alkaloids sum	7.865	0.305	92.184	18.956***	81.552	1.861.	89/90
	Terpenoids richness	18.077	0.143	91.948	10.606***	86.300	0.872	83/86
	Terpenoids sum	18.375	0.028	92.153	12.416***	86.051	0.287	86/88
	Shikimates richness	93.769	0.032	93.576	7.687***	93.330	1.250	80/82
	Shikimates sim	93.009	0.022	92.906	10.921***	92.718	0.866	84/86

The random effect included plots nested in blocks. The table reports the denominator degree of freedom (df), *F*‐values and marginal and conditional *R* ^2^ (marginal before slash and conditional after) for each model (rows). The level of significance is based on FDR‐adjusted *P*‐values and reported with asterisks and dots: *P* < 0.1; *, *P* < 0.05; **, *P* < 0.01; ***, *P* < 0.001. con, conditional; mar, marginal; MC, mycorrhizal colonisation; Shikimates, Shikimates and phenylpropanoid.

As expected, ID explained a notably large amount of variation in plant defence traits and the term was significant for all traits, except leaf toughness (FDR *P*‐value = 0.0727). When ID was included in the fixed effect, the marginal *R* ^2^ ranged from a minimum of 58% for leaf toughness to a maximum of 96% for the terpenoid sum (Table 2). Conversely, plant SR explained only a modest amount of variation in plant defence traits, as indicated by the marginal *R* ^2^ of the models with ID included in the random term, and only plant SR in the fixed term. For these models, the marginal *R* ^2^ was 4% for LMA and 1% for the other significant traits, LDMC, root N and RDMC (Table S6).

**Fig. 5:**
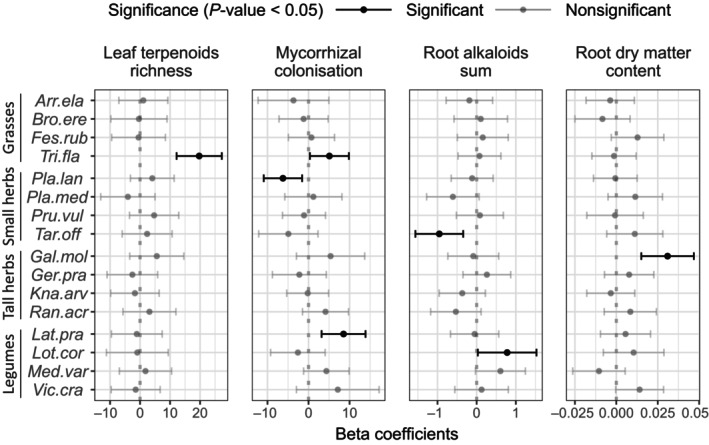
Beta coefficients of plant defence trait changes along the plant species richness gradient (log scale) of the 16 sampled species for leaf terpenoid richness, root mycorrhizal colonisation, root alkaloids sum and root dry matter content. Error bars show a 95% confidence interval. The coefficients, confidence intervals, and *P*‐value were derived from linear mixed models (summarised in Table 2) using the ‘emtrends’ function from the emmeans R package. Only defence trait for which the species richness and species identity interaction were at least marginally significant (false discovery rate (FDR) *P*‐values < 0.1) are reported. Beta coefficients for all defence traits measured in this study are presented in Supporting Information Fig. S2. Species are ordered according to functional groups (grasses, small herbs, tall herbs and legumes). Full species names: Arr.ela, *Arrhenatherum elatius* L.; Bro.ere, *Bromus erectus* Huds.; Fes.rub, *Festuca rubra* L.; Gal.mol, *Galium mollugo* L.; Ger.pra, *Geranium pratense* L.; Kna.arv, *Knautia arvensis* (L.) Coult.; Lat.pra, *Lathyrus pratensis* L.; Lot.cor, *Lotus corniculatus* L.; Med.var, *Medicago × varia* Martyn; Pla.lan, *Plantago lanceolata* L.; Pla.med, *Plantago media* L.; Pru.vul, *Prunella vulgaris* L.; Ran.acr, *Ranunculus acris* L.; Tar.off, *Taraxacum officinale* L.; Tri.fla, *Trisetum flavescens* (L.) P.Beauv.; Vic.cra, *Vicia cracca* L.

Despite the low variance explained by SR, LMA, LDMC and root N responses to SR were independent of ID as the interactions between SR and ID (SR:ID) were not significant (Table 2). This suggests that, at least for these traits, most species responded in the same direction to the plant richness gradient. For RDMC, the interaction term, SR:ID, was marginally significant (FDR *P*‐value 0.0908), suggesting that the response of RDMC to SR partially differed between species (Table 2). Indeed, marginal trend estimation revealed that 9 of the 16 species sampled showed an increase in RDMC along the diversity gradient. This was only significant for one species, *Gallium mollugo*, while the remaining seven species showed neutral or slightly negative trends (Figs 5, S2).

The only other three traits that showed a significant or marginally significant interaction between SR and ID were MC rate (FDR *P*‐value: 0.0173), terpenoid richness in leaves (FDR *P*‐value: 0.0353; Table 2), and the sum of alkaloids in roots (FDR *P*‐value: 0.0908). For MC rate and the sum of alkaloids in roots, species showed negative, neutral and positive responses to SR (Fig. 5). Species richness increased the rate of MC in *Trisetum flavescens* (L.) P.Beauv and *Lathyrus pratensis L*., but decreased it in *Plantago lanceolata*. In addition, SR increased alkaloid production in the roots of *Lathyrus pratensis* but decreased it in the roots of *Taraxacum officinale*. The number of features within terpenoids was mostly neutral among species, with only one species, *Trisetum flavescens*, showing a clear increase in the number of leaf terpenoids (Fig. 5). Overall, these results suggest that while physical defence traits tend to respond similarly among species to the SR gradient, chemical defence traits and MC rate responses to the SR gradient show more species‐specific responses.

## Discussion

This study investigated intraspecific responses of 23 leaf and fine root physical and chemical defence traits to a 19‐yr‐old plant diversity gradient of sixteen grassland plant species. Our main goal was to test whether the accumulation of mutualists and dilution of antagonists, often observed along plant diversity gradients, would promote a reduction in plant defences in high‐diversity communities. In addition, we tested whether this reduction would be stronger in fine root compared with leaf defences, and if it would differ among species. Our results showed that most plant defence traits do not respond to species richness. Furthermore, while defence traits, which are involved in other plant functions, such as resource uptake and usage, responded to species richness similarly among species, chemical defence traits, such as the production of terpenoids, or traits related to the collaboration with mutualists, showed species‐specific responses. Interestingly, some leaf and root defences responded in opposing directions to species richness, suggesting that while changes in resource availability along species richness gradients have a consistent effect among species, they promote the decoupling of defence traits between leaves and fine roots. On the other hand, the species‐specific responses of chemical defence traits and traits related to collaboration with mutualists suggest that the commonly observed dilution of antagonists and accumulation of mutualists across SR gradients may not affect all species equally.

### Effect of plant species richness on leaf defence traits

The leaf defence traits with the most consistent response to the diversity gradient among species were LMA and LDMC. Similar to other studies, these two traits decreased along the diversity gradient (Gubsch *et al*., 2011; Roscher *et al*., 2011b; Lipowsky *et al*., 2015) and are associated with plant defence, against leaf chewers (Caldwell *et al*., 2016), as well as foliar pathogens (Cappelli *et al*., 2020). These traits' responses seem to align with our first hypothesis, that plant defence traits decrease along the plant diversity gradient, due to a reduction in antagonist pressures. However, despite being significant and marginally significant only without correcting for FDR, the production of features within the terpenoids and shikimate and phenylpropanoids pathways in leaves tended to increase with SR in line with the findings of Poeydebat *et al*. (2021). Several phytochemicals within those pathways are known for their defensive role against a variety of leaf antagonists, including arthropods (Dugé de Bernonville *et al*., 2017), pathogens (Lackus *et al*., 2018) and viruses (Zhang *et al*., 2015). This response contradicts our first hypotheses, as it suggests that leaf chemical defences increase with plant diversity.

The opposite responses of these leaf physical and chemical defences to the diversity gradient suggest a trade‐off between physical and chemical defences within species. Despite this general trend, this trade‐off was consistent within all species only for LDMC and leaf terpenoids (Fig. S3). However, the PCA revealed a comparable trade‐off between physical and chemical defences across species in leaf and, to a lower extent, in roots, aligning with findings from other studies (Fernandez‐Conradi *et al*., 2022; Bassi *et al*., 2024). Overall, the finding that physical and chemical defences exhibit a trade‐off within and across species aligns with the growth‐defence trade‐off hypothesis (Lind *et al*., 2013; Zaret *et al*., 2024) as it suggests that plants can simultaneously optimise resource competition and promote defence, as shown in previous studies investigating trade‐offs between constitutive and induced defences (Kempel *et al*., 2011). In our experiment, this trade‐off may arise due to the differential strengths and effects that multiple stressors, such as resource limitation and antagonist pressure, exert on leaf defence traits.

Leaf mass per area and dry matter content have a pivotal role in other plant functions than defence, such as the acquisition of light (Poorter *et al*., 2019). Thus, the response we observed is most likely related to the changes in light availability, as previously detected in our experiment (Roscher *et al*., 2011a; Bachmann *et al*., 2018), a major limiting resource along species richness gradients (Hautier *et al*., 2009), rather than to changes in antagonistic pressure. However, the reduction in LMA and dry matter content, to optimise light‐capturing surface per carbon cost (Poorter *et al*., 2009), inevitably reduces the defensive benefits conferred by these traits. This poses the leaves of plants growing in highly diverse mixtures under a higher risk of antagonist attack. Indeed, even though arthropod herbivore pressure, measured on a biomass or energy flux basis (herbivore biomass or herbivore energy influx divided by plant biomass) was shown to decrease (Ebeling *et al*., 2018; Barnes *et al*., 2020), the proportion of leaf area and leaf biomass eaten by invertebrate herbivores, including gastropods, a group containing many generalists, increases with plant species richness (Meyer *et al*., 2017; but see Seabloom *et al*., 2017). Indeed, at the same site, Bröcher *et al*. (2023) found that the increase or decrease in leaf area eaten by invertebrate herbivores along the species richness gradient was proportional to the change in LDMC, in line with the ‘neighbour contrast susceptibility and defence' hypothesis (Alm Bergvall *et al*., 2006). Given that under light limitation in diverse communities, the relative importance of leaves should increase, and at the same time, the probability of leaf attack increases due to a reduction in physical defence, according to optimal defence theory, plants should promote allocation to defence in leaves (Stamp, 2003). Increasing leaf chemical defences, such as the production of features within the terpenoids and shikimate and phenylpropanoids pathways, could be a cost‐effective way to counterbalance the loss of physical defence without hampering the light‐capturing capacity.

### Effect of plant species richness on root defence traits

Contrary to our first hypothesis, our study indicated that fine roots in high‐diverse mixtures exhibited greater defence than those in low‐diverse mixtures. While RDMC increased, root nitrogen decreased along the plant diversity gradient, suggesting that fine root defences, particularly against root chewers and root‐feeding nematodes (Johnson *et al*., 2010; Moore & Johnson, 2017; Bassi *et al*., 2024), increase along the diversity gradient. However, similar to LMA and LDMC, root nitrogen content and RDMC are related to functions other than plant defences, such as nutrient and water uptake.

The reduction in root nitrogen content along the species richness gradient is consistent with other studies (Mulder *et al*., 2002; van Ruijven & Berendse, 2005) and is probably due to the lower availability of nitrogen, which was previously found in our experiment (Roscher *et al*., 2008), as well as increased productivity and thus nitrogen demand in more diverse communities. Although nitrogen access in diverse mixtures may be enhanced through facilitation effects and resource use complementarity (Bessler *et al*., 2012), our results rather suggest that nitrogen limitation becomes more pronounced in diverse communities. Notably, the experimental plots have not been fertilised since the experiment began, and consistently, higher plant biomass removal from high‐diversity plots (Wagg *et al*., 2022) may have depleted soil nitrogen. Interestingly, the reduction in leaf nitrogen was smaller and not significant, suggesting that the decreased nitrogen content in roots may partly result from reallocation to leaves (Wang *et al*., 2024) to maintain sufficient nitrogen for photosynthesis.

The increased RDMC along the diversity gradient may be attributed to reduced nutrient or water availability (Fischer *et al*., 2019). The simultaneous increase in RDMC and decrease in LDMC may suggest a reallocation of water from the roots to the aboveground part of the plant to support higher biomass and construction of shade leaves in high‐diverse mixtures. A similar opposite response of root and LDMC to light, nutrient and water availability gradients was observed by Freschet *et al*. (2013). However, contrary to root nitrogen content, the observed increase in RDMC is less consistent among species and inconsistent with other studies (Gould *et al*., 2016; Chen *et al*., 2017). Hennecke *et al*. (2025) found no changes in the community‐level root tissue density, a trait strongly related to RDMC (Birouste *et al*., 2014) in the same experiment and at the same time as this study. This suggests that the increase in root dry matter that we observed in our species pool, does not affect the majority or the most dominant species. Nonetheless, the reduction in RDMC of some species is consistent with the findings of Roeder *et al*. (2019, 2021), who found that species with taproots, tend to become older and simultaneously reduce growth rates along the species richness gradient. Thus, in high‐diverse mixtures, some species exhibit a more conservative growth strategy belowground (Bergmann *et al*., 2020), whether this is related to changes in water and nutrient availability or plant age has yet to be determined.

### Decoupled response of leaf and fine root physical defence traits

Contrary to our second hypothesis that fine root defence traits would decrease more than leaf defence traits along diversity gradients, our study revealed that while leaf chemical defences showed a tendency to increase, the few leaf and fine root defence traits that responded to the diversity gradient did so in the opposing direction. These opposite trends between leaf and root defences were driven by traits associated with resource uptake. This suggests a complex relationship between plant defence traits and antagonist pressure, likely mediated by resource availability. While it might be surprising that plants can simultaneously increase root defence and reduce leaf defence, our results contribute to the ongoing debate about whether leaf and root traits are coordinated (Carmona *et al*., 2021; Weigelt *et al*., 2021, 2023; Bueno *et al*., 2023), showing that, at least within species, this may not always be the case. In addition, although other studies have shown that plant diversity has a direct effect on aboveground and belowground antagonist pressures (Ristok *et al*., 2023a,2023b), our study suggests that the observed changes in aboveground and belowground antagonist pressure with species richness may be mostly driven by changes in plant defence traits and not vice versa, as we hypothesised. Combined with the lack of response in several defence traits, our results indicate that the dilution or accumulation of antagonists and mutualists may not occur uniformly (Halliday & Rohr, 2019). Instead, these dynamics may be more complex, differing between aboveground and belowground, as well as among antagonist groups.

Competition for resources in diverse plant communities seems to promote fine root defences. This increase in root defences may explain the reduction in belowground antagonistic pressure from root‐feeding nematodes and arthropods observed in the same experiment (Cortois *et al*., 2017; Dietrich *et al*., 2021; Amyntas *et al*., 2025). However, this increase in defences might also be a response to increased pressure from other groups of antagonists, which have not yet been documented.

Similarly, competition for light reduces leaf physical defences while promoting their chemical defences. This could suggest that under low light availability, plants maintain high chemical defences that, in turn, promote arthropod herbivore and pathogen dilution along the diversity gradient. However, it also raises the possibility that leaf chemical defences may increase due to the accumulation of other antagonist groups, aside from arthropods and leaf pathogens (Ebeling *et al*., 2018; Barnes *et al*., 2020). For example, the observed increase in leaf herbivory rates (Meyer *et al*., 2017; Bröcher *et al*., 2023) could be attributed to damage inflicted by generalist antagonists, such as gastropods, which were not accounted for in other measurements. This may suggest that certain groups of antagonists, particularly generalists, may accumulate rather than dilute along diversity gradients (Keesing *et al*., 2006), thereby explaining the increase in certain defence traits we observed. Further studies are needed to disentangle the complex relationship between plant defence traits, antagonists and mutualists accumulation or dilution.

### Species‐specific response of leaf and fine root defence traits

Plant species identity was the main driver of all defence traits assessed in this study, explaining a substantial proportion of the observed variation. However, species‐specific responses to diversity gradients were less common than anticipated under our third hypothesis. While plant defence traits linked to resource acquisition showed consistent responses across species, species‐specific responses were more frequent in traits with tighter links to defence or collaboration with mutualists. Although only a few traits exhibited this response, root chemical defences showed stronger species‐specific patterns than leaf chemical traits, aligning with the findings of Weinhold *et al*. (2022).

Given the known link between mycorrhiza and the metabolome through the priming of defensive phytochemicals (Frew *et al*., 2022), as well as the association between MC and root chemical defences observed across species in the PCA, it is surprising that our results did not reveal any consistent trends between MC and chemical defence responses to the diversity gradient. Only *T. flavescens* showed a consistent response, with increases in both MC and the number of leaf terpenoids, providing some evidence for priming.

Overall, this species‐specific response may indicate that the dilution of below‐ and aboveground antagonists and the accumulation of belowground mutualists across plant diversity gradients are also species‐specific. Indeed, at the same site, Bröcher *et al*. (2023) found that herbivory rate changes along diversity gradients were species‐specific. These species‐specific responses can be explained by the associational effects, where plant community composition can promote resistance or susceptibility (Barbosa *et al*., 2009; Underwood *et al*., 2014) of focal species to herbivores, depending on whether the focal species is more or less attractive or defended than neighbouring species. While these mechanisms are relatively well understood for aboveground herbivores, it remains unclear to what extent belowground antagonists and mutualists follow the same mechanism. Further studies addressing these issues from a belowground perspective are needed.

### Conclusion

Overall, our results emphasise the complexity of plant defence strategies and their interactions with antagonists across plant diversity gradients. They highlight that plant responses to resource limitation across SR gradients are probably the main drivers of changes in some defence traits, which may in turn influence antagonist pressure. Conversely, chemical defence traits appear to respond to changes in antagonist pressure and, together with traits related to cooperation with mutualists, show more species‐specific responses. This suggests a stronger bottom‐up effect of leaf and fine root physical defence traits on invertebrate herbivores and a top‐down effect of antagonists on leaf chemical defence traits. Finally, our results show that responses of defence traits to plant SR can differ significantly between above‐ and belowground compartments, highlighting the need to integrate both in future studies.

## Competing interests

None declared.

## Author contributions

LB, AW, NMvD, STM and NE designed the study. LB, JH, CA, MDS, AR, YPAdS, AF, VCD, AK, LvS and LW conducted the field campaign and measured leaf and root traits. CA measured the mycorrhizal colonisation rate. RR implemented the bootstrapped CARS‐PLSR model to predict nitrogen content. MZ, SD and VCD performed the untargeted metabolomic analysis. LB performed the statistical analysis and wrote the first draft of the manuscript. All authors contributed to the data interpretation and the revision of each draft.

## Disclaimer

The New Phytologist Foundation remains neutral with regard to jurisdictional claims in maps and in any institutional affiliations.

## Supporting information


**Fig. S1** Interactive sunburst plots showing the numbers of features for the leaf and fine root samples classified with the NPClassisfier.
**Fig. S2** Beta coefficients and 95% confidence intervals depicting the impact of plant species richness (log scale) on leaf and fine root defence traits across the 16 sampled species.
**Fig. S3** Scatter plots showing the standard major axis regression slopes between leaf physical and chemical defence traits within and across species.

**Methods S1** Additional details on the leaf morphological trait measurements.
**Methods S2** Additional details on the nitrogen measuraments.
**Methods S3** Additional details on the untargeted metabolome analysis.
**Table S1** Count of sampled plots across the species richness gradient, the total number of observations and per‐species richness levels for each species.
**Table S2** Literature review of the impact of defensive chemical compounds within the alkaloids, terpenoids, and shikimates and phenylpropanoid pathways on plant antagonists.
**Table S3** Number and percentage of unclassified and classified features in each of the NPClassifier pathways.
**Table S4** Number and percentage of features classified in the NPClassifier superclasses with the highest numbers of features (at least 0.2% of all features).
**Table S5** Loadings of the leaf and root defence traits PCA.
**Table S6** ANOVA table for the linear mixed models with plant defence traits as the response variable and plant species richness (log scale) as predictors.
Please note: Wiley is not responsible for the content or functionality of any Supporting Information supplied by the authors. Any queries (other than missing material) should be directed to the *New Phytologist* Central Office.

## Data Availability

R code, leaf and root trait data, as well as the experimental design metadata, are available through the Jena Experiment database (JExIS) at doi: 10.25829/4VMQ‐V994, and doi: 10.25829/J0YV‐2655, respectively. The near‐infrared spectral data are available at doi: 10.25829/XVJH‐HM95. The raw and processed metabolome data are available from MetaboLights (Yurekten *et al*., 2024; https://www.ebi.ac.uk/metabolights/MTBLS8464); study identifier: MTBLS8464.
