# Supplementary material for: Plant species richness promotes the decoupling of leaf and root defence traits while species‐specific responses in physical and chemical defences are rare
Source: New Phytol. 2025 Feb 27;246(2):729–46. doi: 10.1111/nph.20434 (PMC11923407; doi:10.1111/nph.20434)
Supplement: Supplementary file 1 — Fig. S1 Interactive sunburst plots showing the numbers of features for the leaf and fine root samples classified with the NPClassisfier. Fig. S2 Beta coefficients and 95% confidence intervals depicting the impact of plant species richness (log scale) on leaf and fine root defence traits across the 16 sampled species. Fig. S3 Scatter plots showing the standard major axis regression slopes between leaf physical and chemical defence traits within and across species. [file NPH-246-729-s002.html]

New Phytologist Supporting Information


# New Phytologist Supporting Information

### Article title: Plant species richness promotes the decoupling of leaf and root defence traits while species-specific responses in physical and chemical defences are rare

#### Authors: Leonardo Bassi, Justus Hennecke, Cynthia Albracht, Marcel Dominik Solbach, Akanksha Rai, Yuri Pinheiro Alves de Souza, Aaron Fox, Ming Zeng, Stefanie Döll, Van Cong Doan, Ronny Richter, Anja Kahl, Lea von Sivers, Luise Winkler, Nico Eisenhauer, Sebastian T. Meyer, Nicole M. van Dam & Alexandra Weigelt.

#### Article acceptance date: 13 January 2025

### Figure S1. Interactive sunburst plots showing the number of features for the leaf (left) and fine root (right) samples classified by the NPClassisfier

The inner circle represents individual pathways, the middle circle shows their respective superclasses, and the outer circle shows the classes of features classified by NPClassifier. Colours represent different paths. Hover text gives the total number of features in each category and the percentage of features in that category relative to all features in the tissue.
