# Supplementary material for: Plant species richness promotes the decoupling of leaf and root defence traits while species‐specific responses in physical and chemical defences are rare
Source: New Phytol. 2025 Feb 27;246(2):729–46. doi: 10.1111/nph.20434 (PMC11923407; doi:10.1111/nph.20434)
Supplement: Supplementary file 2 — Methods S1 Additional details on the leaf morphological trait measurements. Methods S2 Additional details on the nitrogen measuraments. Methods S3 Additional details on the untargeted metabolome analysis. Table S1 Count of sampled plots across the species richness gradient, the total number of observations and per‐species richness levels for each species. Table S2 Literature review of the impact of defensive chemical compounds within the alkaloids, terpenoids, and shikimates and phenylpropanoid pathways on plant antagonists. Table S3 Number and percentage of unclassified and classified features in each of the NPClassifier pathways. Table S4 Number and percentage of features classified in the NPClassifier superclasses with the highest numbers of features (at least 0.2% of all features). Table S5 Loadings of the leaf and root defence traits PCA. Table S6 ANOVA table for the linear mixed models with plant defence traits as the response variable and plant species richness (log scale) as predictors. Please note: Wiley is not responsible for the content or functionality of any Supporting Information supplied by the authors. Any queries (other than missing material) should be directed to the New Phytologist Central Office. [file NPH-246-729-s001.pdf]

New Phytologist

New Phytologist Supporting Information

Article title: Plant species richness promotes the decoupling of leaf and root defence traits while species-specific responses in physical and chemical defences are rare

Authors: Leonardo Bassi, Justus Hennecke, Cynthia Albracht, Marcel Dominik Solbach, Akanksha Rai, Yuri Pinheiro Alves de Souza, Aaron Fox, Ming Zeng, Stefanie Döll, Van Cong Doan, Ronny Richter, Anja Kahl, Lea von Sivers, Luise Winkler, Nico Eisenhauer, Sebastian T. Meyer, Nicole M. van Dam & Alexandra Weigelt.

Article acceptance date: 13 January 2025

The following Supporting Information is available for this article:

Table S1 Count of sampled plots across the species richness gradient, the total number of observations and per-species richness levels for each species. 1

Methods S1 Additional details on the leaf morphological trait measurements 1

Methods S2 Additional details on the nitrogen measurements 2

Methods S3 Additional details on the untargeted metabolome analysis 3

Table S2 Literature review of the impact of defensive chemical compounds within the alkaloids, terpenoids, and shikimates and phenylpropanoid pathways on plant antagonists. 5

Table S3 Number and percentage of unclassified and classified features in each of the NPClassifier pathways. 7

Table S4 Number and percentage of features classified in the NPClassifier superclasses with the highest numbers of features (at least 0.2% of all features). 8

Fig. S1 Interactive sunburst plots showing the numbers of features for the leaf (left) and fine root (right) samples classified with the NPClassisfier 9

Table S5 Loadings of the leaf and root defence traits PCA. 10

Table S6 ANOVA table for the linear mixed models with plant defence traits as the response variable and plant species richness (log scale) as predictors. 11

Fig. S2 Beta coefficients and 95% confidence intervals depicting the impact of plant species richness (log scale) on leaf (in green) and fine root (in brown) defence traits across the 16 sampled species. 12

Fig. S3 Scatter plots showing the standard major axis (SMA) regression slopes between leaf physical and chemical defence traits within and across species. 13

References 14

Table S1 Count of sampled plots across the species richness gradient, the total number of observations and per-species richness levels for each species. The table also includes species binomial names, family names, and abbreviations.

Species	Family	Species abbreviation	Species richness levels						Total observations	Total species richness levels
			1	2	4	8	16	60		
Arrhenatherum elatius L.	*Poaceae*	Arr.ela	1	0	2	1	2	2	8	5
Bromus erectus Huds.	*Poaceae*	Bro.ere	1	0	2	2	2	1	8	5
Festuca rubra L.	*Poaceae*	Fes.rub	1	1	0	2	2	1	7	5
Galium mollugo L.	*Rubiaceae*	Gal.mol	1	0	0	1	2	2	6	4
Geranium pratense L.	*Geraniaceae*	Ger.pra	1	0	1	1	2	2	7	5
Knautia arvensis (L.) Coult.	*Caprifoliaceae*	Kna.arv	1	0	2	2	2	2	9	5
Lathyrus pratensis L.	*Fabaceae*	Lat.pra	1	0	1	1	2	2	7	5
Lotus corniculatus L.	*Fabaceae*	Lot.cor	0	1	0	2	2	2	7	4
Medicago x varia Martyn	*Fabaceae*	Med.var	1	1	2	1	2	1	8	6
Plantago lanceolata L.	*Plantaginaceae*	Pla.lan	1	2	2	2	1	2	10	6
Plantago media L.	*Plantaginaceae*	Pla.med	0	1	2	2	2	2	9	5
Prunella vulgaris L.	*Lamiaceae*	Pru.vul	1	0	2	1	2	2	8	5
Ranunculus acris L.	*Ranunculaceae*	Ran.acr	0	2	0	1	2	2	7	4
Taraxacum officinale L.	*Asteraceae*	Tar.off	0	2	1	2	2	2	9	5
Trisetum flavescens (L.) P.Beauv.	*Poaceae*	Tri.fla	0	2	1	2	2	3	10	5
Vicia cracca L.	*Fabaceae*	Vic.cra	1	0	2	1	2	2	8	5

Methods S1 Additional details on the leaf morphological trait measurements

We assessed water repellency (WR; deg.) as an indicator of epicuticular waxes by measuring the average left and right contact angles of a 5 µl water droplet on the adaxial leaf surface (Pérez-Harguindeguy *et al.*, 2013). We collected water droplet images by fixing the leaf on a polystyrene frame with small pins on the side without causing damage and placing a distilled water droplet with a micropipette. After 30 seconds we captured an image of the droplet with a Nikon D5300 camera, a Sigma 18-250 Macro HSM lens, and an additional Rynox macroscopic lens model M-250 in a dark box with backlighting to enhance contrast. We derived contact angles by analysing the images in ImageJ (v. 1.53a; Schneider et al. 2012) using the 'Angle tool'. During the storing or handling process, the leaves of five samples were damaged, resulting in missing water repellency measurements for those samples.

We calculated leaf hair density (N°. of hairs/mm^2) as the ratio of the number of hairs to the leaf area. Leaf images were captured using a camera (Di-Li 2009-16) mounted on a dissecting microscope at 3 to 5X magnification (depending on leaf size) and analysed in ImageJ (v. 1.53a; Schneider et al. 2012). Measurements from the adaxial and abaxial leaf sides were averaged.

We assessed leaf toughness (N/mm) by calculating the ratio of the maximum force required to shear the leaf to its thickness (Pérez-Harguindeguy *et al.*, 2013). We recorded the maximum force to shear on a leaf incision perpendicular and next to the main vein, directed toward the leaf edge, using a Sauter FH 50 dynamometer and a surgical blade type 24. For *Plantago lanceolata* and *Plantago media,* the incision was performed parallel to the main vein. The test was conducted at a consistent speed of 15 mm/min on a motorised vertical test stand. We determined leaf thickness using a digital calliper alongside the incision.

Methods S2 Additional details on the nitrogen measurements

The leaf and fine root subsamples used for the determination of relative nitrogen content (N, % of dry weight) were freeze-dried and ground in a ball mill (MM400, Retsch, Haan, Germany) using a zirconium kit. To prevent excessive heating, the samples were shaken at 30 Hz for 1 minute, followed by cooling at -20°C for 1 to 2 minutes. This cycle was repeated until the samples were transformed into powder. The samples were then freeze-dried again before chemical analysis. Leaf and root nitrogen content was estimated using two methods. For 59% of the samples (150 out of 256 samples), we used 10 mg of tissue material to measure nitrogen content with an elemental analyser (VarioEL II, Elementar, Hanau, Germany; RoMA laboratory of the Max-Planck-Institute for Biogeochemistry in Jena, Germany). For the remaining 41% of the sample, we predicted nitrogen content with a bootstrapped CARS-PLSR model (Richter & Bassi, 2023) using near-infrared spectral data collected with a Multi-Purpose FT-NIR-Analyzer (MPA, Bruker Corporation, Billerica, USA) in transmission mode at 8 cm^{-1} resolution in the 9090 - 4000 cm^{-1} range. Spectra were converted from transmission to absorbance via log10(1/transmission) and transformed using the Standard Normal Variate (SNV) method. The model was calibrated using the hard-measured nitrogen and near-infrared spectral data of this experiment and published data (Bassi *et al.*, 2023) for a total of 238 observations. To calibrate the CARS-PLSR model we randomly drew a set of samples equal in number to the original dataset (with replacement). The remaining samples (out-of-bag samples, about one-third of the total) were used for model validation. This process was repeated 100 times, and the median of the out-of-bag predictions was used to calculate model accuracy indices. The model provided high-accuracy predictions (R2= 98%, RMSE= 0.16%, RPD=7.44). The predicted nitrogen content value from the 100 models were averaged using the median.

Methods S3 Additional details on the untargeted metabolome analysis

Extraction

The untargeted metabolome analysis was performed following the procedure described in Weinhold et al. (2022) with minor modifications (Bassi *et al.*, 2024).

We homogenized the freeze-dried fine root samples manually using scissors, while leaf samples were homogenized in 5 ml tubes using a high-throughput homogenizer HG-600 Geno/Grinder® 2010 (Cole-Parmer, Cambridgeshire, United Kingdom). After a subsequent round of freeze-drying, 10 mg of tissue material was extracted and reduced to powder in 2ml tubes with ceramic beads using a ball mill (Retsch MM400, Haan, Germany) for 8 minutes at 30 Hz. The powdered material was then extracted with 500 µl of buffer comprising 75% v/v methanol (HPLC grade), 25% v/v acetate buffer (2.3 ml acetic acid and 3.41 g ammonium acetate in 1 l of Milli-Q water, pH adjusted to 4.8), and 50 µl of 100 mM IAA-Valin as an internal standard. Subsequently, the samples were homogenized with ceramic beads and a ball mill (Retsch MM400, Haan, Germany) at 30 Hz for 5 minutes. The resulting mixture was centrifuged at 15,000 xg for 15 minutes at room temperature. The supernatant obtained was carefully collected, and the pellet underwent a second extraction with an additional 500 µl of buffer. The two supernatants were then combined and further diluted at a ratio of 1:5 with the extraction buffer. The resulting extracts were stored at −20 °C overnight, followed by a 10-minute centrifugation at 15,000 xg. The clarified supernatant was transferred to HPLC vials.

LC-MS measurements

Chromatographic separations were conducted by injecting 3 µl of the extracts into an UltiMate™ 3000 Standard Ultra-High-Performance Liquid Chromatography system (UHPLC, Thermo Scientific). The system was equipped with an Acclaim RSLC 120 C18 column (150 mm × 2.1 mm, 2.2 µm, ThermoFisher Scientific, Waltham, USA). The flow rate was maintained at 0.4 ml/min, and the column temperature was set to 40 °C. A binary elution gradient was employed with solvent A (water:formic acid 99.9/0.1 in v/v %) and solvent B (acetonitrile:formic acid 99.9/0.1 in v/v %). The gradient profile was as follows: 0–1 min, isocratic 5% B; 1–2 min, linear from 5% to 20% B; 3–8 min, linear from 20% to 25% B; 8–16 min, linear from 25% to 95% B; 16–18 min, isocratic 95% B; 18–18.01 min, linear from 95% to 5% B; 18.01–20 min, isocratic 5% B.

Metabolite detection was conducted using an ESI-UHR-Q-ToF-MS (maXis impact, Bruker Daltonics, Hamburg, Germany), featuring an electrospray ionization source operated in positive mode with data-dependent collision-induced dissociation (Auto-MSMS mode) within the 90-1,600 m/z range. The instrument settings were as follows: Scan range: 90-1,600 m/z; Nebulizer: 2.5 bar; Dry gas flow (nitrogen): 11 l/min; Dry gas temperature: 220 °C; Capillary voltage: 4,500 V; End plate offset: 500 V; Funnel 1 radio frequency (RF): 200 Volts peak-to-peak (Vpp); Funnel 2 RF: 220 Vpp; In-source collision-induced dissociation (CID) energy: 0.0 eV; Hexapole RF: 120 Vpp; Quadrupole ion energy: 4 eV; Quadrupole low mass: 100 m/z; Collision gas: nitrogen; Collision energy: 10 eV; Prepulse storage: 7 µs. For MS/MS fragmentation, the qToF mass spectrometer operated under similar conditions with specific parameters for the collision cell (quadrupole): Collision cell RF: From 400 Vpp to 1,000 Vpp; Transfer time: From 30 to 70 µs; Timing: 50%/50%; Collision energy for MS/MS: 80%; Timing: 50%/50%. Data-dependent CID settings: Intensity threshold: 600; Cycle time: 1 s; Active exclusion: after 2 spectra; Release: after 0.5 min; Smart exclusion: off. Isolation and fragmentation settings: Size- and charge-dependent: width 3–15 m/z; collision energy: 20–30 eV; Charge states included: 1z, 2z, and 3z.

The mass calibration for each chromatogram was executed through an automated infusion at the conclusion of the gradient, employing an HPC mode with a flow rate of 0.1 ml/h. This involved the infusion of a 10 mM

sodium formate cluster of NaOH dissolved in a 50/50 (v/v) mixture of isopropanol and water, containing 0.2% formic acid.

Data processing

The obtained raw data were processed in Bruker Compass MetaboScape 2022b (V. 9.0.1; Build 11878; Bruker Daltonics, Hamburg, Germany). Mass recalibration, peak alignment, peak picking, region complete feature extraction, and grouping of isotopes, adduct, and charge states were done with MetaboScape's T-ReX algorithm. The following settings were used for the peak detection: Intensity threshold: 5000 counts; Minimum peak length: 7 spectra; Feature signal: intensity; Minimum peak length for recursive feature extraction: 7 spectra; Retention time range: 0–18 min; Mass range: 90–1,600 m/z; MS/MS import method: average, grouped by collision energy. The settings for the ion deconvolution were as follows: EIC correlation: 0.8; Primary ion: [M+H]+; Common ions: [M+H-H2O]+; Seed ions: [M+Na]+, [M+K]+; T-ReX-Positive Recalibration: Auto-Detect. We used the following filters: Minimum number of samples: present in 3 of 209; Minimum for recursive feature extraction: present in 3 of 819; Grouping for group filters: species/tissue (n=3), Group filter: feature used when present in at least all samples (100%) of one group. Quality checks were implemented, encompassing the stability of retention time and signal intensity, examination for carry-over, and verification of correct species identity.

Feature filtering

To simplify the SIRIUS pipeline and to maintain comparability between leaf and root samples, raw data processing in Metaboscape was performed on the combined dataset (leaf and root together). This resulted in features with high-intensity values in one tissue type being present at low-intensity values in the other tissue type. While this is not inherently problematic, it does have a significant effect on the sum of the range-scaled intensities as each feature is equally weighted (Sun & Xia, 2024). To reduce the influence of these low-intensity features on our index, we applied the same filtering criteria used in Metaboscape to the leaf and root datasets separately before calculating the index. Features were retained in each tissue type dataset if it reached the intensity threshold of 5000 in at least three replicates (individuals) of a sampling unit.

Table S2 Literature review of the impact of defensive chemical compounds within the alkaloids, terpenoids, and shikimates and phenylpropanoid pathways on plant antagonists. The below table lists the representative chemical compounds, their respective chemical pathways and superclasses, associated plant and its antagonist taxa, and the overall effect of chemical compounds on plant antagonists, extracted from the relevant references.

Pathway	Superclass	Compound	Plant taxa	Antagonist taxa	Effect	Reference
Alkaloids	Ornithine alkaloids	Jacobine and erucifoline	*Jacobaea vulgaris*	*Spodoptera exigua*	Toxic to insects	Nuringtyas et al. 2014
		Scopolamine	*Brugmansia suaveolens*	*Spodoptera frugiperda*	Negative effect on the larvae growth	Alves et al. 2007
	Nicotinic acid alkaloids	Nicotine	*Nicotiana attenuata*	*S. exigua; Trimerotropis* spp.	Defence against insects	Steppuhn et al. 2004
	Tryptophan alkaloids	Terpenoid indole alkaloids	*Catharanthus roseus*	*Manduca sexta*	Defence against insects	Dugé de Bernonville et al. 2017
	Pyrrolizidine alkaloids	Erucifoline and senecionine	*Jacobaea vulgaris*	*Mamestra brassicae*	Defence against insects	Hol *et al.*, 2004
Terpenoids	Monoterpenoids	Monoterpenes	*Populus trichocarpa*; *P. nigra*	*Phytophtora cactorum*	Defence against pathogens	Lackus et al. 2018
	Monoterpenoids	Iridoid glycosides	*Plantago lanceolata*	*Diaporthe adunca*; *Fusarium moniliforme*	Defence against pathogens	Marak *et al.*, 2002
	Apocarotenoid	α-Ionone	*Solanum lycopersicum*	*Frankliniella occidentalis*; *Spodoptera litura*	Inducing plant resistance	Murata et al. 2019
	Steroids	Brassinosteroid	*Nicotiana tabacum; Oryza sativa*	*Pseudomona syringae*; *Oidium* spp; *Magnaporthe grisea*; *Xanthomonas oryzae*	Disease resistance	Nakashita et al. 2003
			Arabidopsis thaliana	*Cucumber mosaic virus*	Virus stress tolerance	Zhang et al. 2015
		Holaphyllamine	*A. thaliana*	*P. syringae*	Resistance to pathogen	Zahid et al. 2017

Table S2 continues.

Table S2 Continued.

Pathway	Superclass	Compound	Plant taxa	Antagonist taxa	Effect	Reference
Shikimates and phenylpropanoid	Phenolic acids	Total soluble phenol	*Zea mays*	*S. frugiperda*	Insect resistance	Zhang et al. 2022
		Phenols	*Solanum melongena*	*Leucinodes orbonalis*	Resistance to herbivores	Prasannalaxmi and Rani 2016
	Flavonoids	Flavonoid	*Solanum lycopersicum*	*Bemisia tabaci*; *Orius sauteri*	herbivore-induced direct and indirect defences	Yang et al. 2023
		Flavonoids	*Vaccinium myrtillus*	*Paraphaeosphaeria*; *Botrytis cinerea*	Early defence against biotic stress	Koskimäki et al. 2009
		Flavonoids	*Sorghum bicolor*	*S. frugiperda*	Resistance against fall armyworm	Grover *et al.*, 2022
	Coumarins	Coumarins	*Daphne laureola*	*Pseudenargia ulicis*; *Noctua janthe*; *Spodoptera littoralis*	Variability in the responses of herbivores	Alonso et al. 2009
		Coumarins	*Helianthus annuus*	*Puccinia helianthin*	Systemic acquired resistance	Prats et al. 2002
	Stilbenoids	Stilbenes	*Vitis vinifera*; *Vitis candicans*	*Botrytis cinerea*; *Plasmopara viticola*; *Erysiphe necator*	Defence priming	Olivier et al. 2018
	Diarylheptanoids	Phenylphenalenone phytoalexins	*Musa* spp.	*Radopholus similis*	Resistance to the burrowing nematode	Hölscher et al. 2014
		Oregonin	*Alnus rubra*	*Trichoplusia ni*; *Orgyia leucostigma*; *Hyphantria cunea*; *Malacosoma californicum*	Protecting red alder from leaf-eating insects	Lea et al. 2021

Table S3 Number and percentage of unclassified and classified features in each of the NPClassifier pathways. The table reports a summary of the entire dataset and leaf and fine root separately. The total number of features and their percentage over the full dataset detected in leaf, fine root and their combination is reported. The pathways of classified features are ordered ascendingly based on the number of features. Pathways in bold were used for the main analysis. Unclassified = features that were classified but did not meet our accuracy threshold (ZODIAC score and NPC pathway probability above 0.5). Unknown = features not classified by SIRIUS.

	**Number of features**			**Percentage of features (%)**		
**NPC pathway**	**Leaf**	**Fine root**	**Full dataset**	**Leaf**	**Fine root**	**Full dataset**
**Shikimates and Phenylpropanoids**	**2131**	**2149**	**2173**	**20**	**20**	**20**
**Terpenoids**	**1597**	**1607**	**1618**	**15**	**15**	**15**
Fatty acids	332	330	334	3	3	3
Amino acids and Peptides	306	304	306	3	3	3
Polyketides	270	276	276	3	3	3
**Alkaloids**	**252**	**257**	**258**	**2**	**2**	**2**
Carbohydrates	221	222	222	2	2	2
Unclassified	1574	1592	1615	15	15	15
Unknown	3758	3788	3865	35	36	36
Total	10441	10525	10667			

Table S4 Number and percentage of features classified in the NPClassifier superclasses with the highest numbers of features (at least 0.2% of all features). The table reports a summary of the entire dataset and leaf and fine root separately. The total number of features and their percentage over the full dataset detected in leaf, fine root and their combination is reported. The pathways of classified features are ordered ascendingly based on the number of features. Pathways in bold were used for the main analysis.

		Number of features			Percentage of features (%)		
**NPC pathway**	**NPC superclass**	**Leaf**	**Fine root**	**Full dataset**	**Leaf**	**Fine root**	**Full dataset**
**Shikimates and phenylpropanoids**	**Flavonoids**	813	812	836	7.62	7.61	7.84
	**Phenylpropanoids (C6-C3)**	402	402	402	3.77	3.77	3.77
	**Lignans**	221	221	221	2.07	2.07	2.07
	**Phenolic acids (C6-C1)**	197	198	198	1.85	1.86	1.86
	**Coumarins**	187	188	188	1.75	1.76	1.76
	**Isoflavonoids**	111	124	124	1.04	1.16	1.16
	**Phenylethanoids (C6-C2)**	72	72	72	0.67	0.67	0.67
**Terpenoids**	**Triterpenoids**	860	861	864	8.06	8.07	8.10
	**Sesquiterpenoids**	214	217	217	2.01	2.03	2.03
	**Monoterpenoids**	148	148	148	1.39	1.39	1.39
	**Diterpenoids**	115	121	122	1.08	1.13	1.14
	**Apocarotenoids**	101	99	102	0.95	0.93	0.96
	**Steroids**	87	90	93	0.82	0.84	0.87
	**Meroterpenoids**	22	22	22	0.21	0.21	0.21
Fatty acids	Glycerolipids	114	112	114	1.07	1.05	1.07
	Fatty Acids and Conjugates	71	71	71	0.67	0.67	0.67
	Octadecanoids	35	35	35	0.33	0.33	0.33
	Glycerophospholipids	25	25	25	0.23	0.23	0.23
Amino acids and peptides	Small peptides	212	210	212	1.99	1.97	1.99
	Amino acid glycosides	50	50	50	0.47	0.47	0.47
Polyketides	Polycyclic aromatic polyketides	58	59	59	0.54	0.55	0.55
	Naphthalenes	32	32	32	0.30	0.30	0.30
	Coumarins	24	24	24	0.22	0.22	0.22
	Cyclic polyketides	23	24	24	0.22	0.22	0.22
	Macrolides	22	22	22	0.21	0.21	0.21
**Alkaloids**	**Pseudoalkaloids**	41	42	42	0.38	0.39	0.39
	**Ornithine alkaloids**	35	35	35	0.33	0.33	0.33
	**Tyrosine alkaloids**	30	30	31	0.28	0.28	0.29
	**Anthranilic acid alkaloids**	25	25	25	0.23	0.23	0.23
	**Small peptides**	22	22	22	0.21	0.21	0.21
Carbohydrates	Saccharides	108	108	108	1.01	1.01	1.01
	Nucleosides	59	59	59	0.55	0.55	0.55
	Polyols	22	22	22	0.21	0.21	0.21

Fig. S1 Interactive sunburst plots showing the numbers of features for the leaf (left) and fine root (right) samples classified with the NPClassisfier.

Please see the attached SI file.

Table S5 Loadings of the leaf and root defence traits PCA. The top section presents results for leaf traits and the bottom section for root traits. Log-transformed variables are indicated. Traits negatively correlated with defence (nitrogen and specific root length; Table 1) were multiplied by -1 to ensure that positive loadings consistently represent increased defence. The proportion of variance explained by each principal component is reported in the last row of each section.

		**PC1**	**PC2**	**PC3**	**PC4**
Leaf	Water repellency	**0.4404**	0.1728	-0.1853	**0.3318**
	Hair density	0.1137	0.2686	**0.7716**	0.1090
	Toughness (log)	**0.4168**	-0.2073	0.0984	**0.4382**
	Leaf mass per area (log)	-0.2448	**-0.3729**	**0.4195**	0.3127
	Dry matter content	**0.4464**	-0.2656	-0.1145	0.2625
	-[Nitrogen] (log)	-0.2505	**-0.5720**	0.0084	-0.0362
	Alkaloids sum (log)	**-0.3647**	**0.3560**	0.1168	**0.4319**
	Terpenoids sum (log)	-0.1874	**0.3952**	-0.2934	0.2803
	Shikimates sum	**-0.3595**	-0.1882	-0.2680	**0.5030**
	Explained Variance (%)	34.99	20.41	12.45	8.85

		**PC1**	**PC2**	**PC3**	**PC4**
Fine root	-[Specific root length] (log)	0.3315	**0.5134**	-0.0771	0.1138
	Diameter (log)	**0.3596**	**0.4135**	-0.3048	0.1006
	Mycorrhizal colonisation	**0.4123**	-0.1413	-0.0467	**-0.8053**
	Dry matter content (log)	0.0396	**0.5772**	0.3096	0.0628
	-[Nitrogen] (log)	**-0.3644**	0.1298	**0.5906**	-0.0708
	Alkaloids sum	**0.4545**	-0.1943	0.2176	0.1845
	Terpenoids sum (log)	0.3288	**-0.3942**	0.0590	**0.5291**
	Shikimates sum (log)	**0.3765**	-0.0485	**0.6352**	-0.0743
	Explained Variance (%)	42.20	28.04	12.70	6.38

Table S6 ANOVA table for the linear mixed models with plant defence traits as the response variable and plant species richness (log scale) as predictors. The random effect included plot nested in block and plant species identity as random intercept or random intercept and slope (only for MC and Leaf terpenoid richness). The table reports the denominator degree of freedom (df), F-value, P-value, marginal and conditional R^2, Beta-coefficient (model slope), its standard error and lower and upper limit of the 95% confidence interval for each model (rows). Traits with a significant (P < 0.05) and marginally significant (P < 1) response to the plant species richness gradient are highlighted in bold and underlined respectively. Abbreviations: Shikimates = Shikimates and phenylpropanoid, MC = Mycorrhizal colonisation.

		df	F-value	P-value	Marginal R^2 (%)	Conditional R^2 (%)	Beta-coefficient	Standard error	Lower CI	Upper CI
Leaf	Water repellency	104.188	0.006	0.9377	0	86	-0.0705	0.9004	-1.8537	1.7127
	Hair density	32.932	0.450	0.5070	0	79	-0.0762	0.1136	-0.3010	0.1486
	Toughness	110.103	0.081	0.7760	0	59	0.0139	0.0486	-0.0823	0.1100
	**Leaf mass per area**	**23.443**	**19.778**	**0.0002**	**4**	**85**	**-1.8052**	**0.4059**	**-2.6088**	**-1.0017**
	**Dry matter content**	**27.030**	**12.476**	**0.0015**	**1**	**91**	**-0.0050**	**0.0014**	**-0.0079**	**-0.0022**
	Nitrogen	15.137	4.047	0.0624	0	89	-0.0507	0.0252	-0.1006	0.0008
	Alkaloids richness	25.154	0.199	0.6595	0	91	-0.0927	0.2079	-0.5043	0.3189
	Alkaloids sum	109.87	0.196	0.6589	0	87	0.0336	0.0758	-0.1166	0.1837
	Terpenoids richness	22.980	1.394	0.2499	0	97	2.0379	1.7263	-1.3801	5.4559
	**Terpenoids sum**	**110.179**	**4.150**	**0.0440**	**0**	**96**	**0.7213**	**0.3541**	**0.0203**	**1.4222**
	Shikimates richness	28.728	0.095	0.7602	0	96	0.3484	1.1309	-1.8902	2.5870
	Shikimates sum	32.843	3.972	0.0546	1	93	1.2992	0.6519	0.0088	2.5897
Fine root	Specific root length	28.342	0.023	0.8813	0	73	-0.2197	1.4577	-3.1064	2.6670
	Diameter	108.106	0.474	0.4928	0	78	-0.0037	0.0054	-0.0143	0.0069
	MC	16.182	0.630	0.4389	0	84	0.8907	1.1224	-1.3363	3.1177
	**Dry matter content**	**108.173**	**6.261**	**0.0138**	**1**	**72**	**0.0049**	**0.0020**	**0.0010**	**0.0089**
	**Nitrogen**	**109.22**	**13.452**	**0.0004**	**1**	**90**	**-0.0695**	**0.0189**	**-0.1070**	**-0.032**
	Alkaloids richness	28.906	0.684	0.4149	0	83	0.3180	0.3845	-0.4431	1.0792
	Alkaloids sum	11.650	0.323	0.5805	0	89	-0.0485	0.0854	-0.2175	0.1205
	Terpenoids richness	15.628	0.161	0.6938	0	86	-1.6789	4.1854	-9.9651	6.6072
	Terpenoids sum	22.493	0.016	0.8995	0	89	0.1727	1.3518	-2.5035	2.8489
	Shikimates richness	109.110	0.167	0.6835	0	82	0.9110	2.2287	-3.5014	5.3233
	Shikimates sum	108.538	0.008	0.9303	0	87	0.0679	0.7739	-1.4643	1.6000

Fig. S2 Beta coefficients and 95% confidence intervals depicting the impact of plant species richness (log scale) on leaf (in green) and fine root (in brown) defence traits across the 16 sampled species. The coefficients, confidence intervals, and p-value were derived from linear mixed models (summarised in Table 1) using the 'emtrends' function from the 'emmenas' R package. Significant coefficients ($p < 0.05$) are shown in darker colours. Abbreviation: Shikimates = Shikimates and phenylpropanoid. Species are ordered according to functional groups (grass, small herbs, tall herbs and legumes).

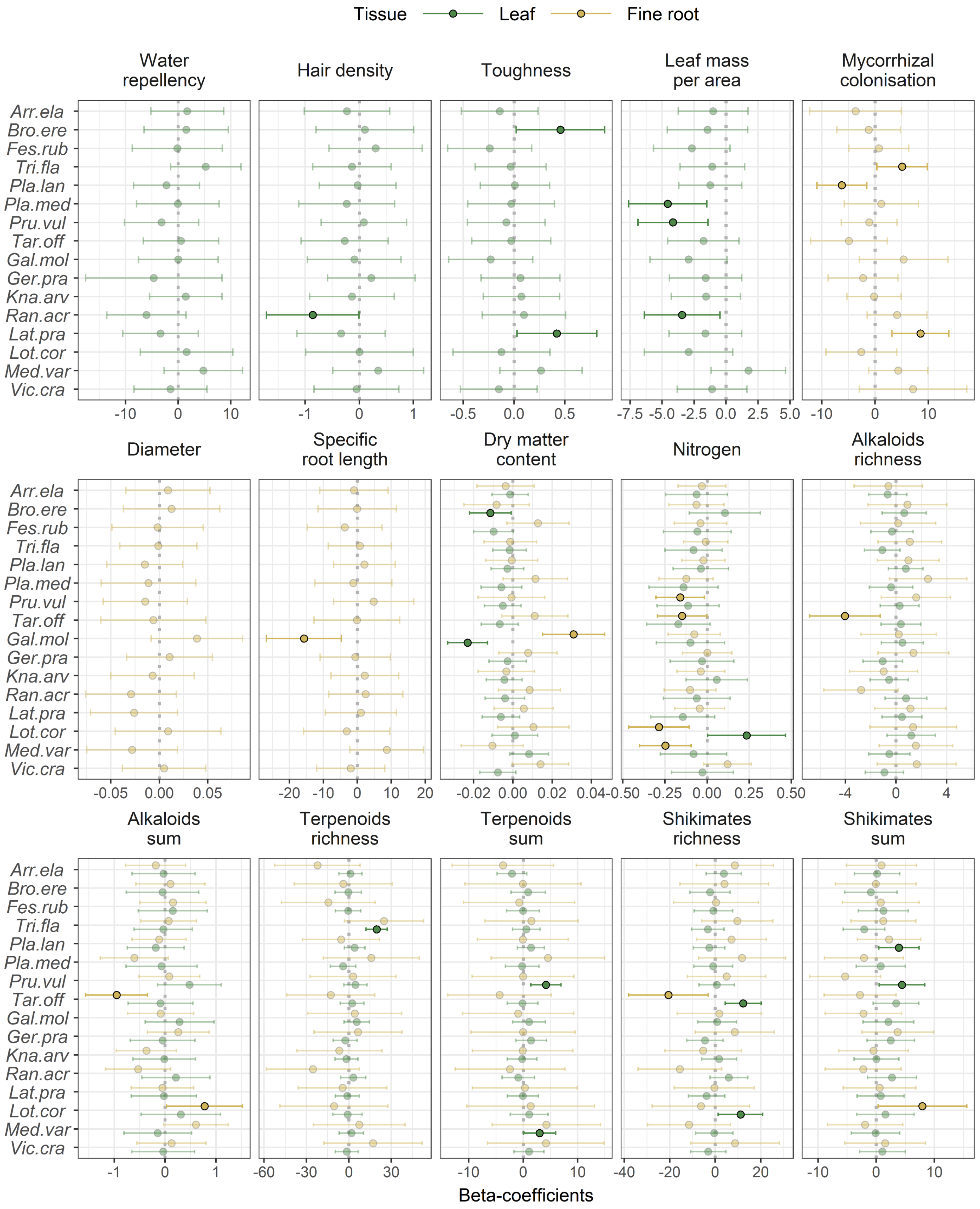

Fig. S3 Scatter plots showing the standard major axis (SMA) regression slopes between leaf physical and chemical defence traits within and across species. Black lines represent slopes across species, while grey lines represent slopes within species. Significant slopes are depicted with solid lines, whereas non-significant slopes are shown as dashed lines. Within each panel, the significance level of the slopes across species and the significance level of the interaction with species identity is reported. The significance levels of the SMA regressions were calculated using the smatr R package. Only leaf physical and chemical defence traits that showed a trade-off in the PCA (Fig. 3, Table S5) and responded significantly or marginally significantly to the diversity gradient (Fig. 3) were included. Leaf toughness, LMA (leaf mass per area), and terpenoids were log-transformed. Significance levels are indicated as follows: *** for P < 0.001, ** for P < 0.01, * for P < 0.05.

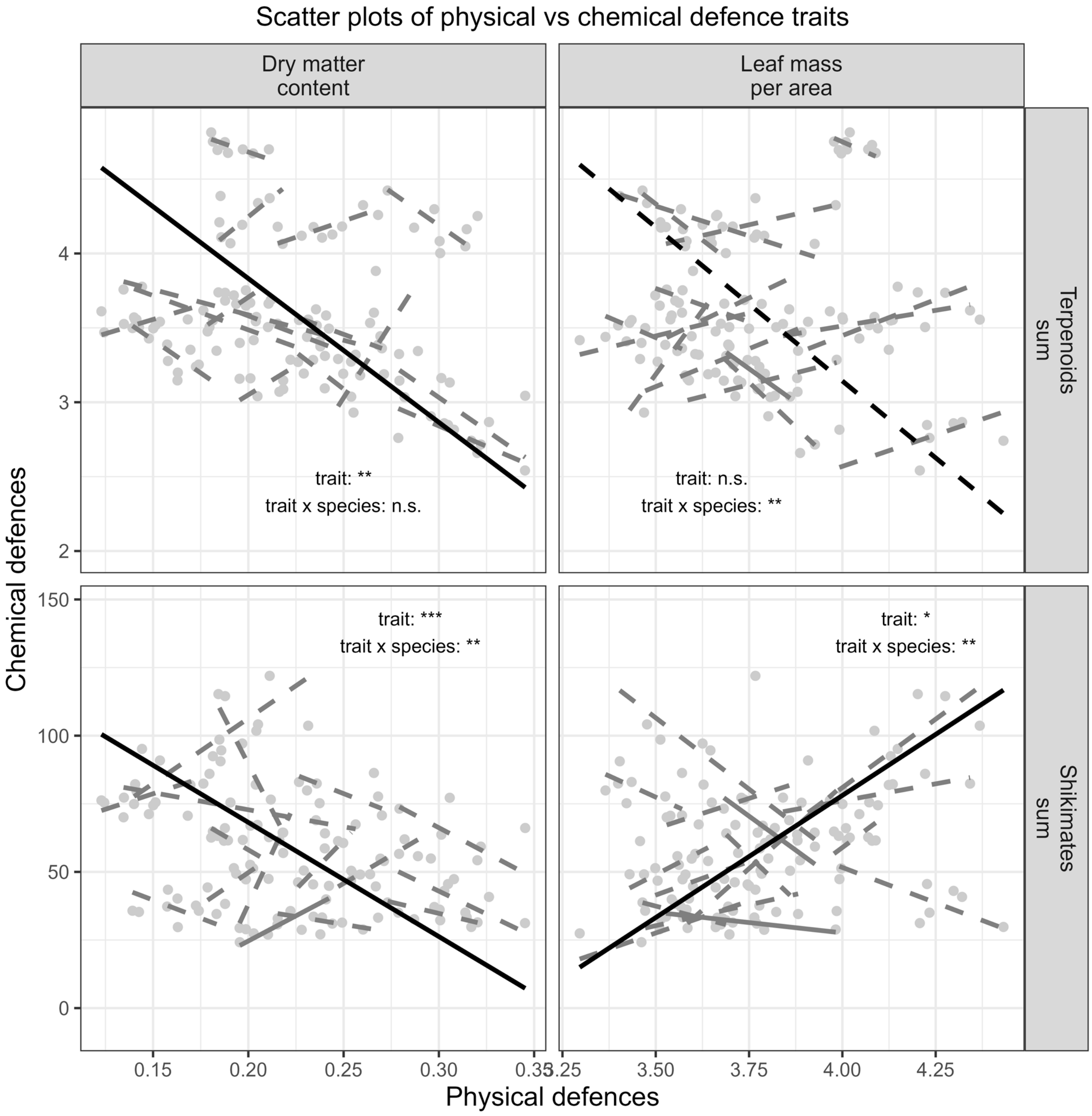

References

Alonso C, García IM, Zapata N, Pérez R. **2009**. Variability in the behavioural responses of three generalist herbivores to the most abundant coumarin in Daphne laureola leaves. *Entomologia Experimentalis et Applicata* **132**: 76–83.

Alves MN, Sartoratto A, Trigo JR. **2007**. Scopolamine in Brugmansia suaveolens (Solanaceae): Defense, allocation, costs, and induced response. *Journal of Chemical Ecology* **33**: 297–309.

Bassi L, Hennecke J, Albracht C, Bröcher M, Solbach MD, Schaller J, Doan VC, Wagner H, Eisenhauer N, Ebeling A, *et al.* 2024. Uncovering the secrets of monoculture yield decline: trade-offs between leaf and root chemical and physical defence traits in a grassland experiment. *Oikos* **2024**: e10061.

Bassi L, Patzak R, van Dam N, Weigelt A. **2023**. Bassi, L., Patzak, R., van Dam, N. and Weigelt, A. 2023. Leaf and fine root NIRS Spectra from old and new (dBEF) monoculture plots in 2020 (Version 17) [Data set]. *Jena Experiment Information System*: doi: https://doi.org/10.25829/7ASR-PZ67.

Dugé de Bernonville T, Carqueijeiro I, Lanoue A, Lafontaine F, Sánchez Bel P, Liesecke F, Musset K, Oudin A, Glévarec G, Pichon O, *et al.* 2017. Folivory elicits a strong defense reaction in Catharanthus roseus: metabolomic and transcriptomic analyses reveal distinct local and systemic responses. *Scientific Reports* **7**: 40453.

Grover S, Shinde S, Puri H, Palmer N, Sarath G, Sattler SE, Louis J. **2022**. Dynamic regulation of phenylpropanoid pathway metabolites in modulating sorghum defense against fall armyworm. *Frontiers in Plant Science* **13**: 1–14.

Hol GW., Macel M, van Veen JA, van der Meijden E. **2004**. Root damage and aboveground herbivory change concentration and composition of pyrrolizidine alkaloids of Senecio jacobaea. *Basic and Applied Ecology* **5**: 253–260.

Hölscher D, Dhakshinamoorthy S, Alexandrov T, Becker M, Bretschneider T, Buerkert A, Crecelius AC, De Waele D, Elsen A, Heckel DG, *et al.* 2014. Phenalenone-type phytoalexins mediate resistance of banana plants (Musa spp.) to the burrowing nematode Radopholus similis. *Proceedings of the National Academy of Sciences of the United States of America* **111**: 105–110.

Koskimäki JJ, Hokkanen J, Jaakola L, Suorsa M, Tolonen A, Mattila S, Pirttilä AM, Hohtola A. **2009**. Flavonoid biosynthesis and degradation play a role in early defence responses of bilberry (Vaccinium myrtillus) against biotic stress. *European Journal of Plant Pathology* **125**: 629–640.

Lackus ND, Lackner S, Gershenzon J, Unsicker SB, Köllner TG. **2018**. The occurrence and formation of monoterpenes in herbivore-damaged poplar roots. *Scientific Reports* **8**: 17936.

Lea CS, Bradbury SG, Constabel CP. **2021**. Anti-Herbivore Activity of Oregonin, a Diarylheptanoid Found in Leaves and Bark of Red Alder (Alnus rubra). *Journal of Chemical Ecology* **47**: 215–226.

Marak HB, Biere A, van Damme JMM. **2002**. Two herbivore-deterrent iridoid glycosides reduce the in-vitro growth of a specialist but not of a generalist pathogenic fungus of Plantago lanceolata L. *Chemoecology* **12**: 185–192.

Murata M, Kobayashi T, Seo S. **2019**. α-Ionone, an Apocarotenoid, Induces Plant Resistance to Western Flower Thrips, Frankliniella occidentalis, Independently of Jasmonic Acid. *Molecules* **25**: 17.

Nakashita H, Yasuda M, Nitta T, Asami T, Fujioka S, Arai Y, Sekimata K, Takatsuto S, Yamaguchi I, Yoshida S. **2003**. Brassinosteroid functions in a broad range of disease resistance in tobacco and rice. *The Plant Journal* **33**: 887–898.

Nuringtyas TR, Verpoorte R, Klinkhamer PGL, van Oers MM, Leiss KA. **2014**. Toxicity of Pyrrolizidine Alkaloids to Spodoptera exigua Using Insect Cell Lines and Injection Bioassays. *Journal of Chemical Ecology* **40**: 609–616.

Olivier V, Spring J-L, Gindro K. **2018**. Stilbenes: biomarkers of grapevine resistance to fungal diseases. *OENO One* **52**: 235–241.

Pérez-Harguindeguy N, Díaz S, Garnier E, Lavorel S, Poorter H, Jaureguiberry P, Bret-Harte MS, Cornwell WK, Craine JM, Gurvich DE, *et al.* **2013**. New handbook for standardised measurement of plant functional traits worldwide. *Australian Journal of Botany* **61**: 167–234.

Prasannalaxmi K, Rani PU. **2016**. Interactions between herbivore Leucinodes orbonalis G. and its host plant Solanum melongena L.: A study on insect induced direct plant responses. *Allelopathy Journal* **37**: 273–286.

Prats E, Rubiales D, JorrÍn J. **2002**. Acibenzolar- S -methyl-induced resistance to sunflower rust (Puccinia helianthi) is associated with an enhancement of coumarins on foliar surface. *Physiological and Molecular Plant Pathology* **60**: 155–162.

Schneider CA, Rasband WS, Eliceiri KW. **2012**. NIH Image to ImageJ: 25 years of image analysis. *Nature Methods* **9**: 671–675.

Steppuhn A, Gase K, Krock B, Halitschke R, Baldwin IT. **2004**. Nicotine's Defensive Function in Nature. *PLoS Biology* **2**: e217.

Sun J, Xia Y. **2024**. Pretreating and normalizing metabolomics data for statistical analysis. *Genes & Diseases* **11**: 100979.

Weinhold A, Döll S, Liu M, Schedl A, Pöschl Y, Xu X, Neumann S, Dam NM. **2022**. Tree species richness differentially affects the chemical composition of leaves, roots and root exudates in four subtropical tree species. *Journal of Ecology* **110**: 97–116.

Yang F, Shen H, Huang T, Yao Q, Hu J, Tang J, Zhang R, Tong H, Wu Q, Zhang Y, *et al.* **2023**. Flavonoid production in tomato mediates both direct and indirect plant defences against whiteflies in tritrophic interactions. *Pest Management Science* **79**: 4644–4654.

Zahid A, Jaber R, Laggoun F, Lehner A, Remy-Jouet I, Pamlard O, Beaupierre S, Leprince J, Follet-Gueye M-L, Vicré-Gibouin M, *et al.* **2017**. Holaphyllamine, a steroid, is able to induce defense responses in Arabidopsis thaliana and increases resistance against bacterial infection. *Planta* **246**: 1109–1124.

Zhang D-W, Deng X-G, Fu F-Q, Lin H-H. **2015**. Induction of plant virus defense response by brassinosteroids and brassinosteroid signaling in Arabidopsis thaliana. *Planta* **241**: 875–885.

Zhang H, Hu Z, Luo X, Wang Y, Wang Y, Liu T, Zhang Y, Chu L, Wang X, Zhen Y, *et al.* **2022**. ZmRop1 participates in maize defense response to the damage of Spodoptera frugiperda larvae through mediating ROS and soluble phenol production. *Plant Direct* **6**: 1–13.
